# Cannabinoids activate the insulin pathway to modulate mobilization of cholesterol in *C*. *elegans*

**DOI:** 10.1371/journal.pgen.1010346

**Published:** 2022-11-08

**Authors:** Bruno Hernandez-Cravero, Sofia Gallino, Jeremy Florman, Cecilia Vranych, Philippe Diaz, Ana Belén Elgoyhen, Mark J. Alkema, Diego de Mendoza

**Affiliations:** 1 Laboratorio de Fisiología Microbiana, Instituto de Biología Molecular y Celular de Rosario (IBR), CONICET, Facultad de Ciencias Bioquímicas y Farmacéuticas, Universidad Nacional de Rosario, Rosario, Argentina; 2 Laboratorio de Fisiología y Genética de la Audición, Instituto de Investigaciones en Ingeniería Genética y Biología Molecular "Dr. Héctor N. Torres" (INGEBI), CONICET, Buenos Aires, Argentina; 3 Department of Neurobiology, University of Massachusetts Medical School, Worcester, Massachusetts, United States of America; 4 Department of Biomedical and Pharmaceutical Sciences, University of Montana, Missoula, Montana, United States of America; Vanderbilt University Medical Center, UNITED STATES

## Abstract

The nematode *Caenorhabditis elegans* requires exogenous cholesterol to survive and its depletion leads to early developmental arrest. Thus, tight regulation of cholesterol storage and distribution within the organism is critical. Previously, we demonstrated that the endocannabinoid (eCB) 2-arachidonoylglycerol (2-AG) plays a key role in *C*. *elegans* since it modulates sterol mobilization. However, the mechanism remains unknown. Here we show that mutations in the *ocr-2* and *osm-9* genes, coding for transient receptors potential V (TRPV) ion channels, dramatically reduce the effect of 2-AG in cholesterol mobilization. Through genetic analysis in combination with the rescue of larval arrest induced by sterol starvation, we found that the insulin/IGF-1signaling (IIS) pathway and UNC-31/CAPS, a calcium-activated regulator of neural dense-core vesicles release, are essential for 2-AG-mediated stimulation of cholesterol mobilization. These findings indicate that 2-AG-dependent cholesterol trafficking requires the release of insulin peptides and signaling through the DAF-2 insulin receptor. These results suggest that 2-AG acts as an endogenous modulator of TRPV signal transduction to control intracellular sterol trafficking through modulation of the IGF-1 signaling pathway

## Author summary

Although cannabis extracts have been used in folklore medicine for centuries, the past few years have seen an increased interest in the medicinal uses of cannabinoids, the bioactive components of the cannabis plant, for treatment of many diseases of the nervous system. However, the human body naturally produces endocannabinoids that are similar to the cannabinoids present in *Cannabis sativa*. Our goal is to understand how endocannabinoids maintain cholesterol homeostasis in animals, underscoring the importance of cholesterol balance for healthy life. Both cholesterol excess and cholesterol deficiency can have detrimental effects on health, and a myriad of regulatory processes have thus evolved to control the metabolic pathways of sterol metabolism. The nematode *C*. *elegans* is auxotroph for sterols. Therefore, in contrast to mammals, they cannot synthesize sterols and dietary supply is essential for survival. The aim of our study was to elucidate the mechanism by which endocannabinoids abolish larval arrest of *C*. *elegans* induced by cholesterol depletion. We show that endocannabinoids stimulate the insulin pathway, which affects development, reproduction and life span, to modulate mobilization of cholesterol in *C*. *elegans*. Our studies have important implications for a better understanding of human pathological conditions associated with impaired cholesterol homeostasis.

## Introduction

Cholesterol is essential for a diverse range of cellular processes, including hormone signaling, fat metabolism, and membrane structure and dynamics. Dysregulation of cholesterol and lipid homeostasis can have a major impact on development and disease [1,2]. Cholesterol deficiency can result in blunt steroid hormone production, reduced serotonin levels, vitamin deficiencies, and increased mortality, whereas cholesterol excess is a risk factor for cardiovascular disease, diabetes, neurodegeneration, and inflammation [3–8]. Thus, understanding cholesterol and lipid homeostasis is critical to illuminate aspects of human health and longevity.

The nematode *Caenorhabditis elegans* requires exogenous cholesterol since it cannot synthesize it de novo [9]. In *C*. *elegans*, cholesterol regulates at least two processes. First, it is required for growth and progression through larval stages, as well as for proper shedding of old cuticles during larval molting events [10]. Second, it regulates entry into a specialized diapause stage adapted for survival under harsh conditions, called the dauer larva [11]. Tight spatial and temporal regulation of uptake, storage, and transport of sterols to appropriate subcellular compartments is required for cholesterol to exert its diverse cellular functions [10].

Despite the pivotal role of sterols in *C*. *elegans* development, the regulation of cholesterol metabolism is only now beginning to be understood. Previously, it was shown that worms grown in the absence of cholesterol arrest as dauer-like larvae in the second generation [9]. The sterols that govern dauer formation are bile-acid-like hormones called dafachronic acids (DAs) [11]. Molecular mechanisms underlying their function have been intensely studied. DAs inhibit dauer formation by binding to the nuclear hormone receptor DAF-12, which, in the absence of DAs, activates the dauer program [12,13]. Even though cholesterol is associated with cell membranes and interacts with multiple lipid species, very little is known about how lipids influence cholesterol trafficking. It was recently discovered that the *C*. *elegans* glycolipids phosphoethanolamine glucosylceramides (PEGCs) stimulate the growth of worms by as yet unknown mechanism under conditions of cholesterol scarcity [14]. We also recently reported that the best studied endocannabinoids (eCBs), 2-arachidonoyl glycerol (2-AG) and arachidonoyl ethanolamine (AEA), lipid messengers that elicit a plethora of biological functions in mammals, enhance traffic of cholesterol in *C*. *elegans* [15]. We found that these eCBs stimulate worm growth under conditions of cholesterol scarcity and reverse the developmental arrest of *ncr-2; ncr-1* mutants [15]. *ncr-1* and *ncr-2* encode proteins with homology to the human Niemann-Pick type C (NP-C) disease gene (*NPC-1*) and are involved in intracellular cholesterol trafficking in *C*. *elegans* [16]. The mechanism by which these signaling lipids exert their effects on cholesterol homeostasis within large endocrine networks is unknown. Here we show that 2-AG promotes cholesterol mobilization through pathways that are independent of known *C*. *elegans* cannabinoid-like receptors that mediate regulation of regenerative axon navigation [17] and behavior [18]. We find that mutations in the *ocr-2* and *osm-9* genes, coding for transient receptors potential of the vanilloid subtype (TRPV) ion channels, dramatically reduce the effect of 2-AG in cholesterol mobilization. We also show that the IIS pathway and UNC-31/CAPS, the calcium-activated regulator of dense-core vesicles exocytosis (DCVs), are necessary for 2-AG-mediated stimulation of cholesterol mobilization. This suggests that 2-AG-dependent cholesterol traffic requires signaling of insulin peptides through the DAF-2 insulin receptor. Our results indicate that 2-AG acts as an endogenous modulator of TRPV channels to control intracellular sterol trafficking through modulation of the IIS signaling pathway.

## Results

### FAAH-4 is involved in cholesterol homeostasis in *C*. *elegans*

**Fig 1:**
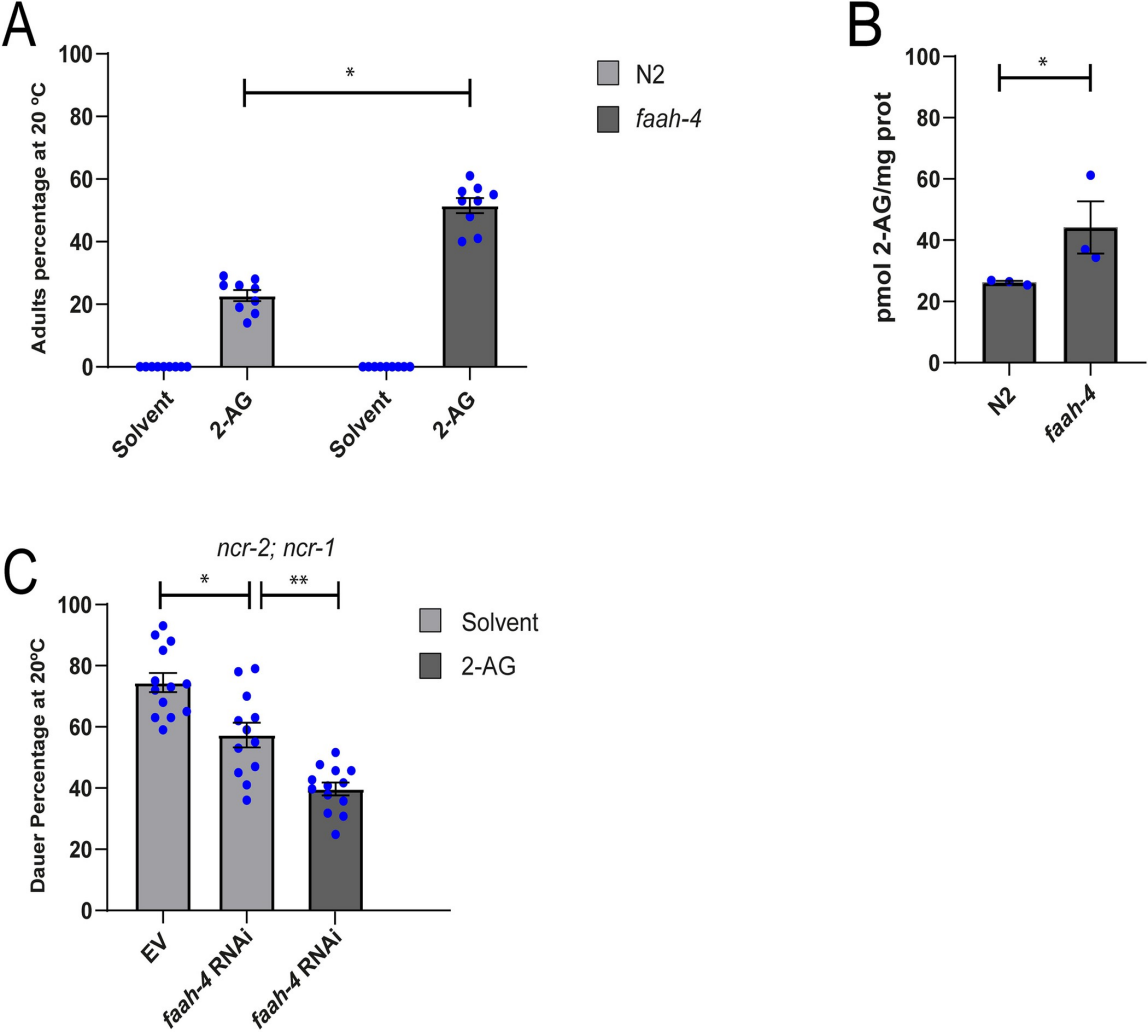
FAAH-4 is involved in cholesterol mobilization by 2-AG. (A) Wild type and *faah-4* animals grown for two generations in the absence of cholesterol at 20°C arrest as L2*. Feeding with 2-AG (50 μg/ml) increases the formation of adults in *faah-4* animals compared with N2 animals. All Pairwise are Multiple Comparison Procedures (Dunn’s Method), *p < 0.05. All values are from n = 3 independent experiments are shown as Mean ± SEM. (B) Intracellular levels of 2-AG are elevated in *faah-4* animals. T-test, *p < 0.005. All values are from n = 3 independent experiments shown as Mean ± SEM. (C) *faah-4* RNAi reduces the dauer formation of strain *ncr-2; ncr-1* and enhances the ability of low concentrations of 2-AG (10 μM) to suppress the *daf-c* phenotype of these animals. All Pairwise are Multiple Comparison Procedures (Holm-Sidak method), *p < 0.002, **p < 0.001. All values are from n ≥ 3 independent experiments are shown as Mean ± SEM. N2 is the *C*. *elegans* wild-type strain.

*C*. *elegans* interrupt reproductive development and arrest as L2-like larvae (L2*) when grown for two generations without cholesterol [9]. Previously, we found that this arrest is partially abolished by supplementation with the eCB 2-AG [15, Fig 1A]. Recent work has revealed that the monoacylglycerol lipase FAAH-4, but no other *C*. *elegans* FAAH enzymes, hydrolyzes 2-AG [19]. To test the specificity of eCB signaling we asked whether a 6951 pb deletion of the *faah-4* gene (*Δfaah-4*) [19] could enhance the rescuing effect of 2-AG on the arrest induced by cholesterol depletion in *C*. *elegans*. Indeed, we found that under sterol free conditions exogenous 2-AG (50 μM) significantly increases the formation of adults in *Δfaah-4* compared to wild-type animals (Fig 1A). In agreement with this result, *faah-4* animals displayed elevated levels of endogenous 2-AG compared to the wild type (Fig 1B, [19]). Next, we investigated whether *faah-4* deficiency relieves the phenotype of mutations that perturb cholesterol transport. We found that FAAH-4 RNAi decreased the Daf-c penetrance in null mutants for the Niemann-Pick homologues *ncr-2; ncr-1* (Fig 1C). As expected, FAAH-4 RNAi also enhanced the ability of a low concentration of 2-AG (10 μM) to partially suppress dauer formation of *ncr-2; ncr-1* double mutants (Fig 1C). These results suggest that FAAH-4 is an important enzyme in cholesterol homeostasis.

### 2-AG controls cholesterol homeostasis through NPR-19 and NPR-32 independent pathways

**Fig 2:**
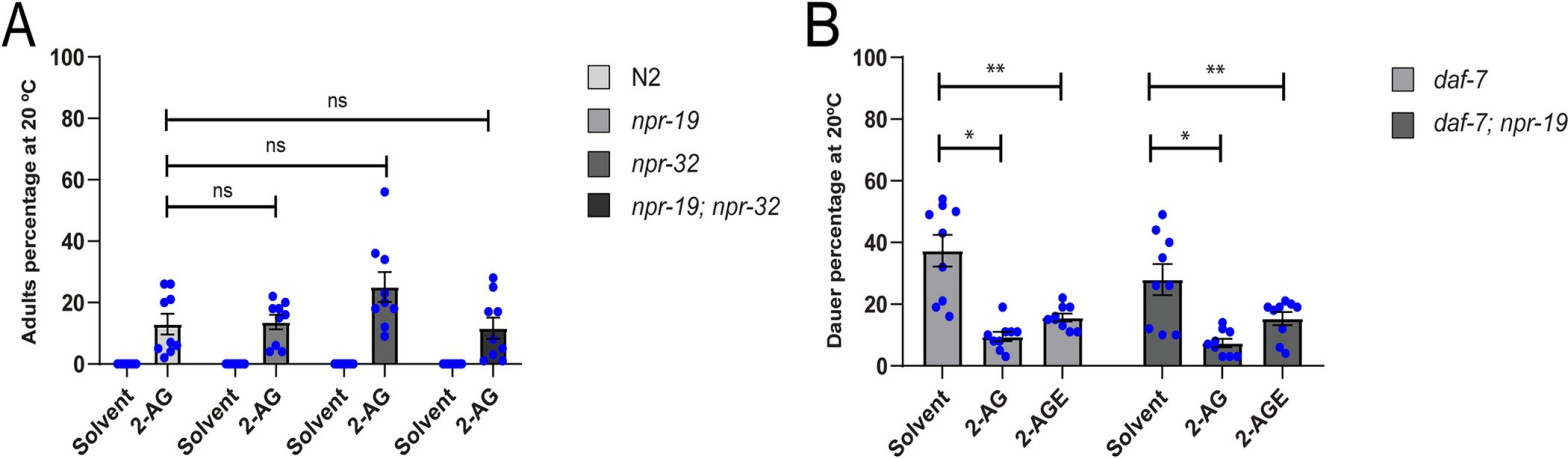
Endocannabinoids stimulate transport of cholesterol by employing pathways independent of *npr-19* and *npr-32*. (A) 2-AG suppresses L2* larval arrest of *npr-19*, *npr-32 and npr-19; npr-32* animals grown for two generations on plates containing 0 μg/ml of cholesterol. All values are from n = 3 independent experiments shown as Mean ± SEM. ns = not significant. N2 is the *C*. *elegans* wild-type strain. (B) Endocannabinoids suppress dauer arrest of *daf-7* and *daf-7; npr-19* grown on plates containing 5 μg/ml of cholesterol. Mann-Whitney rank sum test, *p < 0.001, **p < 0.001. All values are from n = 3 independent experiments are shown as Mean ± SEM. The concentration of 2-AG was 50 μM and of 2-AGE 100 μM.

The biological effects of 2-AG in mammals are mediated through its interaction with the G-protein coupled type-1 (CB1) and type-2 (CB2) cannabinoid receptors [20–22]. In a *C*. *elegans* genome study where vertebrate CB1 were compared to other G protein-coupled receptors (GPCRs), the neuropeptide receptors (NPRs) NPR-19 and NPR-32, were shown to have conservation of the critical amino acid residues involved in eCB ligand binding [17]. NPR-19 has been shown to be a 2-AG receptor that modulates monoaminergic (e.g., serotonin and dopamine) signaling in *C*. *elegans* [18]. To determine whether NPR-19 is required for the 2-AG-mediated stimulation of cholesterol mobilization, we screened mutant animals for loss of 2-AG-dependent enhancement of cholesterol trafficking. We found that 2-AG relieved the L2* arrest of *npr-19* and *npr-32* mutants when cholesterol was depleted from the diet (0 μg/ml) (Fig 2A), indicating that 2-AG stimulation of cholesterol trafficking is independent of NPR-19 and NPR-32. Moreover, 2-AG partially rescued the arrest of the double mutant *npr-19*; *npr-32* under cholesterol depletion (Fig 2A), ruling out the possibility that these two receptors might redundantly participate in the eCB effect. We also tested the interaction of NPR-19 with the DAF-7/TGF-β pathway, which plays an important role in promoting development by affecting cholesterol metabolism and DA production [14]. *daf-7* mutants constitutively form dauer larvae when grown in normal dietary cholesterol (13 μM) due to impaired cholesterol trafficking [14]. We found that *npr-19* did not increase the penetrance of the daf-c defects seen in *daf-7* mutants at 20°C (Fig 2B). Moreover, dauer formation in both *daf-7* and *daf-7*; *npr-19* mutant animals were rescued by either 2-AG or 2-arachidonoylglycerol-eter (2-AGE) (Fig 2B), a non-hydrolysable analog of 2-AG. Together, these data demonstrate that 2-AG acts in cholesterol mobilization through NPR-19 and NPR-32 independent pathways. We also tested several 2-AG receptor candidates, such as GPCRs and neurotransmitter receptors, identified by examination of protein BLAST data using human CB1 receptor sequences. However, animals mutated in these potential targets were rescued by 2-AG under cholesterol depletion growing conditions (S1 Table).

### TRPV channels are required for 2-AG-dependent cholesterol mobilization

**Fig 3:**
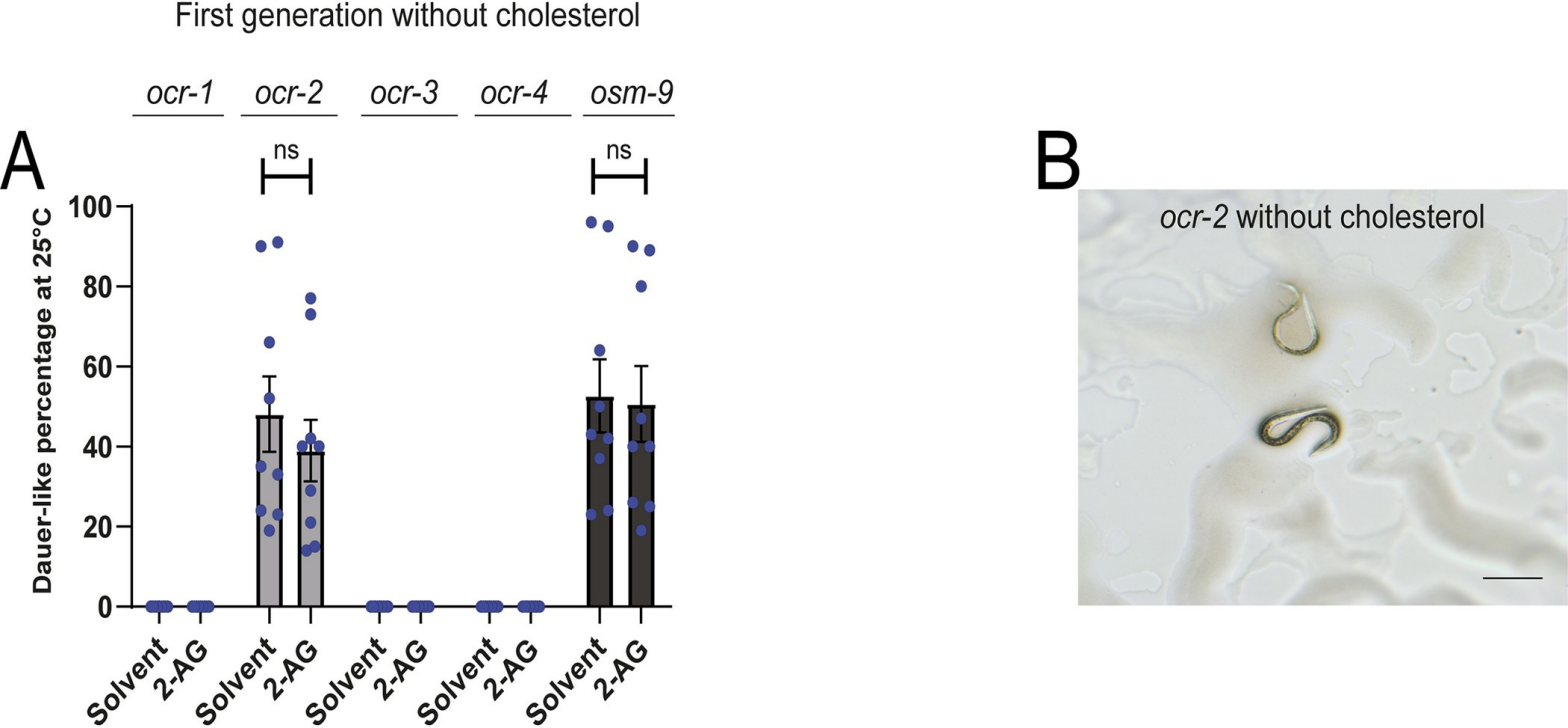
*ocr-2 and osm-9* are required for 2-AG-dependent cholesterol mobilization. (A) Worms were grown in media with 0 μg/ml of cholesterol during one generation at 25°C. All values are from n = 3 independent experiments shown as Mean ± SEM. ns = not significant. (B) *ocr-2* undergoes dauer-like formation in the first generation when grown at 25°C in cholesterol-free medium. The black straight line represents 0.25mm.

In mammals many cannabinoids can activate TRPV channels [23]. *ocr-2* encodes a channel of the TRPV subfamily that functions in *C*. *elegans* olfaction, nociception and osmosensation [24]. We found that, similar to *C*. *elegans fat-3* and *fat-4* mutants, which are deficient in PUFAs and display aberrant cholesterol mobilization [15], *ocr-2* animals displayed a high incidence of arrested dauer-like larvae in the first generation without cholesterol (Fig 3A and 3B). While 2-AG partially suppresses dauer formation of *fat* mutants [15], 2-AG was unable to rescue the arrest of *ocr-2* animals starved from cholesterol. To determine the specificity of *ocr-2* requirement for mobilization of cholesterol, we examined mutants for other TRPV channels. The *C*. *elegans* genome encodes five members of the TRPV family, *ocr-1* through *ocr-4* and *osm-9*. We found that null mutations in *ocr-1*, *ocr-3* and *ocr-4* produced almost 100% first generation gravid adults in the absence of cholesterol (Fig 3A). In contrast, and similar to *ocr-2* mutants, about 50% of cholesterol starved *osm-9* animals exhibited a dauer-like phenotype in the first generation (Fig 3A and 3B). 2-AG was also unable to prevent the arrest of *osm-9* animals starved from cholesterol (Fig 3A). Taken together, these data demonstrate that both *osm-9* and *ocr-2* mutations eliminate 2-AG-dependent cholesterol mobilization.

In *C*. *elegans*, both, *osm-9* and *ocr-2* TRPV genes are expressed in ADF serotonergic neurons and also co-expressed in five pairs of non-serotonergic chemosensory neurons: the AWA, ADL, ASH in the head and the PHA and PHB in the tail [25]. It has been reported that OSM-9 and OCR-2 regulate serotonin (5-HT) biosynthesis in ADF neurons through the modulation of the *tph-1* gene expression, which encodes a key enzyme required for 5-HT biosynthesis from tryptophan [26]. A recent report suggested that 2-AG stimulates 5-HT release through a pathway requiring OSM-9, from the serotonergic ADF neurons to modulate *C*. *elegans* behavior [27]. However, we found that the ability of 2-AG to rescue the development of cholesterol depleted worms is unaffected by mutations in *tph-1* or in 5-HT receptor mutants (S1 Fig and S1 Table). These results indicate that 2-AG stimulation of cholesterol mobilization in *C*. *elegans* is independent of 5-HT release.

Both OSM-9 and OCR-2 are thought to function cooperatively and assemble into homo- and hetero-tetramers [28]. Recent electrophysiological studies employing the *Xenopus laevis* oocyte expression system demonstrated that OSM-9/OCR-2 respond to warming, suggesting that these channels cooperatively function as a temperature receptor [29]. Because OSM-9 and OCR-2 channels mediate influx of divalent cations with a preference for calcium [30], we hypothesized that 2-AG might be an agonist of channels containing OSM-9 and/or OCR-2 subunits and that activation of the channel is the trigger mechanism for 2-AG-induced mobilization of cholesterol in *C*. *elegans*. To test this hypothesis, we expressed OSM-9 and OCR-2 in *Xenopus* oocytes. Our analysis shows that 2-AG did not elicit currents in *Xenopus* oocytes injected with *osm-9* and *ocr-2* cRNAs either alone or in combination (S2 Fig). However, warm stimulus (about 36°C) evoked currents in *Xenopus* oocytes injected with both *osm-9* and *ocr-2* cRNAs (S2 Fig), indicating the expression of active channels.

To determine whether 2-AG can induce neuronal activity in vivo in neurons that co-express *osm-9* and *ocr-2* we used calcium imaging. 100 μM 2-AG failed to induce calcium transients in animal that express the GCaMP6 in ASH neurons (S3 Fig). The fact that 2-AG did not induce OSM-9/OCR-2 channel activation *in vitro* or *in vivo*, suggests that this compound acts upstream or in parallel of TRPV channels in sensory transduction.

### The cannabinoid based T-Type calcium channel blocker NMP331 antagonizes the action of 2-AG on cholesterol mobilization

**Fig 4:**
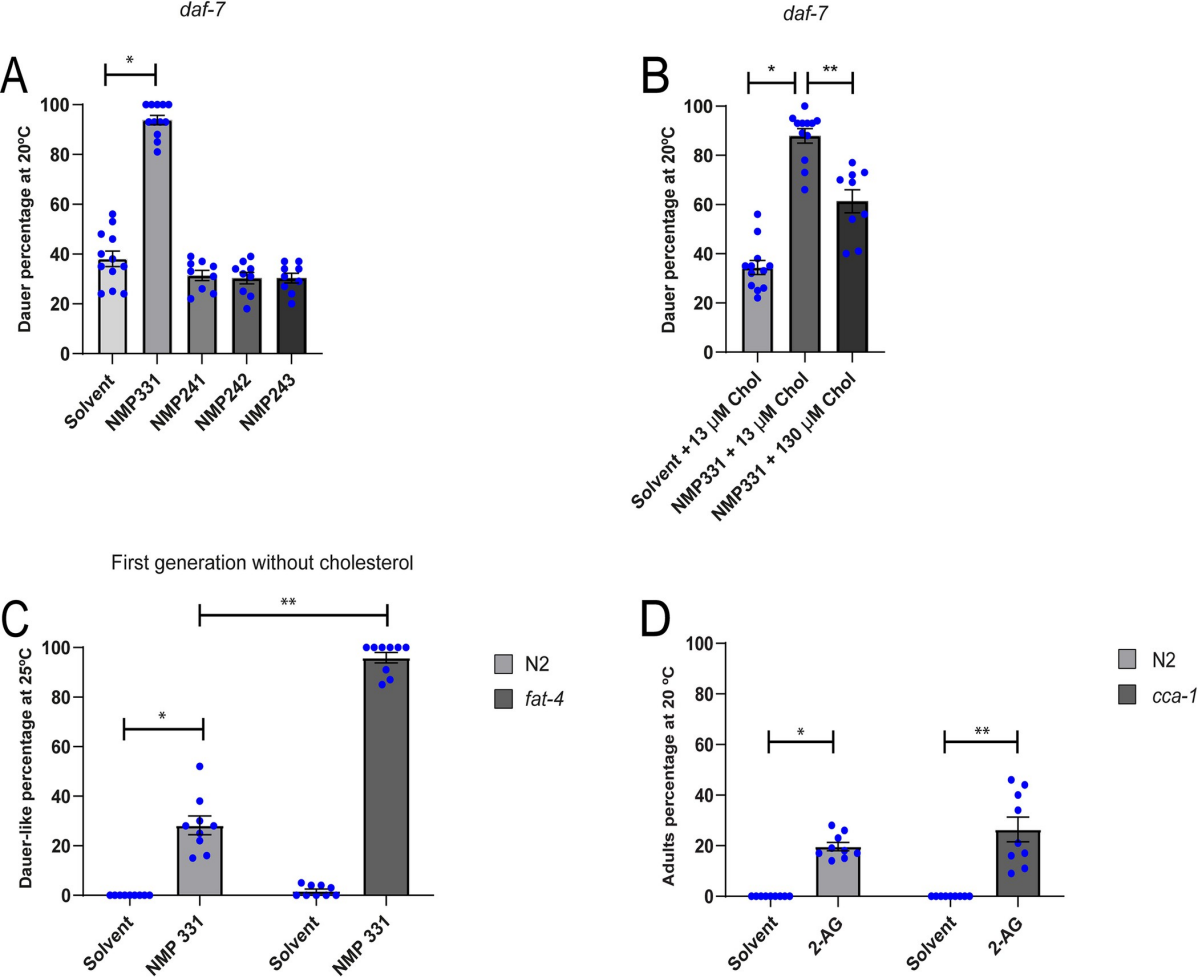
The cannabinoid receptor antagonist NMP331 induces dauer formation. (A) The dauer arrest of *daf-7* worms is augmented by NMP331. All pairwise are multiple comparison procedures (Holm-Sidak method), *p < 0.001. All values are from n ≥ 3 independent experiments are shown as Mean ± SEM. (B) Dauer arrest induced by NMP331 can be rescued by high cholesterol diet. All Pairwise are Multiple Comparison Procedures (Holm-Sidak method), *p < 0.001. **p < 0.001. All values are from n ≥ 3 independent experiments shown as Mean ± SEM. (C) N2 and *fat-4* animals undergo a dauer-like arrest induced by NMP331 in the first generation when grown in cholesterol free medium. Mann-Whitney rank sum test, *p < 0.001. **p < 0.005. All values are from n = 3 independent experiments are shown as Mean ± SEM. The concentration of NMP331 was 50 μM. (D) The 2-AG mediated suppression of L2* arrest of N2, grown for two generations in medium devoid of cholesterol, is independent of CCA-1. Mann-Whitney rank sum test, *p < 0.001. t-test, **p < 0.001. All values are from n = 3 independent experiments shown as Mean ± SEM. N2 is the *C*. *elegans* wild-type strain.

Since the effect of 2-AG on cholesterol mobilization appears not to be mediated by CB receptor orthologues (Fig 2), we tested synthetic cannabinoid ligands with the expectation that some of these compounds might act as inverse agonists/antagonists of 2-AG. This approach has been used to identify lipophilic molecules that interact with putative eCB receptors with conserved function but which diverge from canonical mammalian receptors [31]. We reasoned that if a synthetic ligand blocks a cannabinoid signaling pathway involved in cholesterol trafficking, the Daf-c defects seen in *daf-7* mutants would be increased. We tested whether a series of CB1/CB2 receptor ligands (NMP compounds) that target both CB receptors and T-type calcium channels [32,33] modulate the Daf-c phenotype of *daf-7* (e1372) mutants. Of the four compounds tested, only one compound, NMP331 (named as compound 10 in reference 33), at a concentration of 1 μM robustly enhanced the dauer phenotype of *daf-7* at a semi-permissive temperature (20°C) (Fig 4A). Supplementation of growth media with an excess of cholesterol lowered dauer formation in *daf-7* exposed to NMP331 (Fig 4B), suggesting that this compound affects sterol mobilization. If NMP331 affects cholesterol mobilization, we predicted that, in the presence of this compound, wild-type animals would arrest already in the first generation in the absence of externally provided sterols. While wild type animals produced almost 100% gravid adults in the first generation without cholesterol, exposure to NMP331 in the absence of cholesterol resulted in a high incidence (20%) of arrested dauer-like larvae (Fig 4C). Furthermore, 2-AG antagonized the enhancing effect of 1 μM of NMP331 on dauer formation in *daf-7* worms in a dose-response manner (S4A Fig).

Strikingly, when *fat-4* animals, which are unable to synthesize AEA or 2-AG [15], were exposed to NMP331 in a cholesterol depleted medium, formed 90% dauer-like worms in the first generation, while untreated animals produced mostly gravid adults (Fig 4C).

Using radio-ligand assays and electrophysiology in human embryonic kidney cells, it was determined that compound NMP331, in addition to showing high affinity for CB1 receptors, also blocks CaV3.2 T-type calcium channels [33]. Unlike vertebrates that possess three genes that encode T-calcium channels, the genome of *C*. *elegans* encodes a single T-type channel named *cca-1* [34]. We found that 2-AG-dependent cholesterol mobilization was still present in a *cca-1* mutant (Fig 4D), suggesting that this T-type channel is not the target of NMP331. We also found that NMP331 can still exert its effects in *npr-19* and *npr-32* mutants (S4B and S4C Fig). This result shows that NMP331 impairs cholesterol mobilization trough NPR-19 and NPR-32 independent pathways.

Taken together we conclude that NMP331 impacts cholesterol availability, transport and/or metabolism by antagonizing the stimulatory role of 2-AG in cholesterol mobilization. At this point we do not know whether NMP331 affects other molecular targets, such as ion channels, involved in the cannabinoid-mediated modulation of cholesterol trafficking.

### SBP-1 is required for 2-AG modulation of cholesterol mobilization

To identify potential *C*. *elegans* genes controlling the regulatory circuit of 2-AG-mediated cholesterol mobilization, we performed RNAi enhancer screen on *daf-7* temperature sensitive mutants, using an RNAi library containing transcription factors, transporters and nuclear receptors potentially involved in cholesterol homeostasis (S2 Table). We first surveyed for enhancement of the *daf-7* Daf-c phenotype and, in a secondary survey, screened for loss of 2-AG-dependent rescue of the developmental arrest caused by cholesterol depletion.

**Fig 5:**
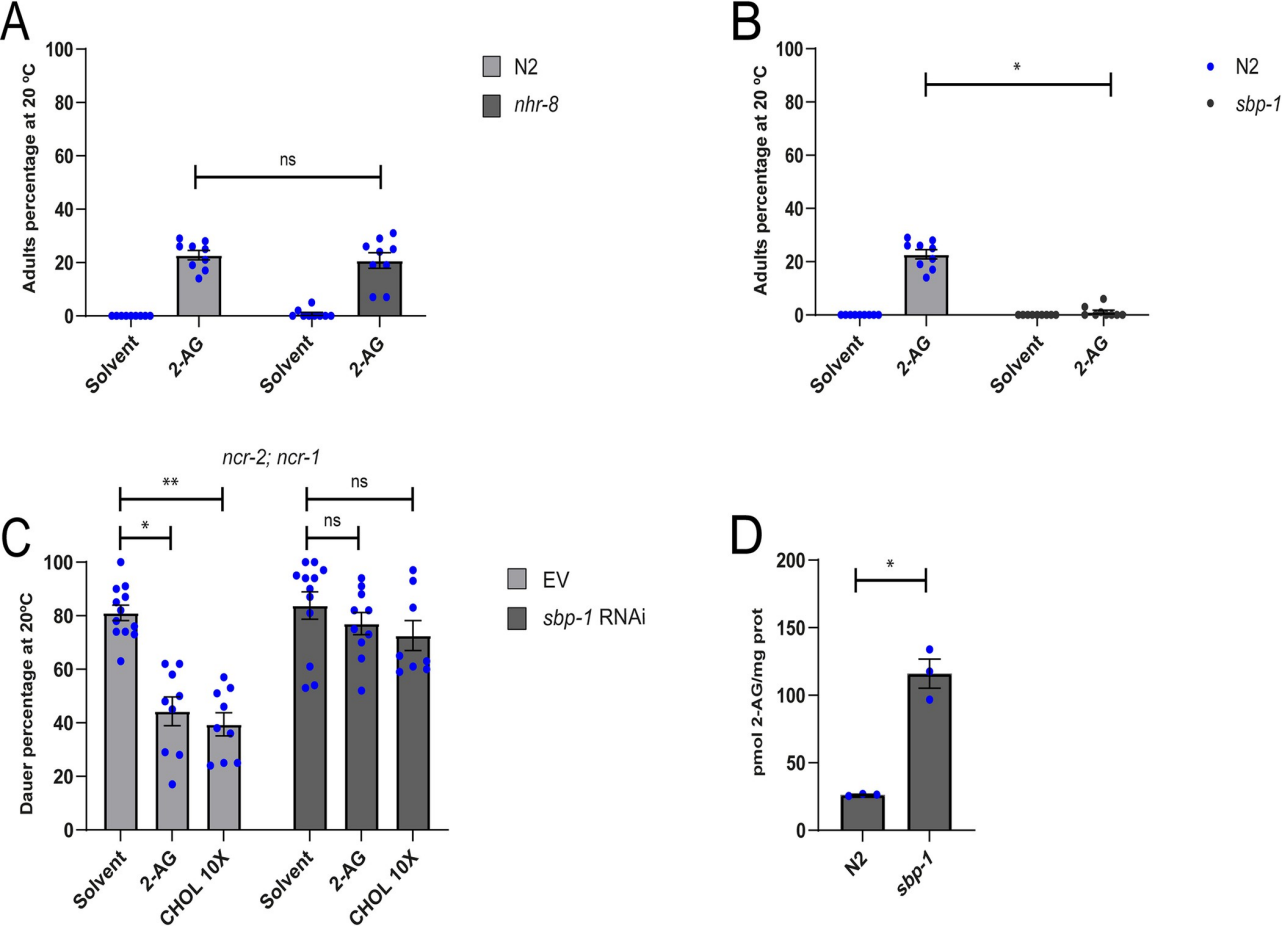
SBP-1 is required for 2-AG modulation of cholesterol mobilization. (A) 2-AG rescue the L2* arrest of *nhr-8* worms grown for two generations under 0 μg/ul of cholesterol at 20°C. All values are from n = 3 independent experiments shown as Mean ± SEM. ns = not significant. (B) 2-AG is unable to rescue the L2* arrest of *sbp-1* grown for two generations under 0 μg/ml cholesterol at 20°C. All Pairwise are Multiple Comparison Procedures (Dunn’s Method), *p < 0.001. All values are from n = 3 independent experiments are shown as Mean ± SEM. (C) 2-AG is unable to suppress dauer formation of *a ncr-2; ncr-1* strain exposed to *sbp-1* RNAi. Kruskal-Wallis One-way analysis of variance on ranks, *p < 0.001, **p < 0.001. All values are from n ≥ 3 independent experiments are shown as Mean ± SEM. ns = not significant. (D) 2-AG levels are elevated in *sbp-1* animals. t-test, *p < 0.005. All values are from n = 3 independent experiments are shown as Mean ± SEM. N2 is the C. elegans wild-type strain.

We identified two loci, *nhr-8* and *sbp-1*, that in combination with *daf-7* give a strong Daf-c constitutive phenotype (S2 Table). *nhr-8*, codes for a nuclear receptor that plays an important role in cholesterol homeostasis in *C*. *elegans* [35], while *sbp-1*, encodes the single orthologue of the sterol regulatory element (SREBP) family which regulates transcription of genes required in many aspects of lipid metabolism [36]. We found that supplementation with 2-AG partially rescued the developmental arrest of *nhr-8* animals depleted of cholesterol (Fig 5A). Thus, *nhr-8* is not positioned within the 2-AG pathway of cholesterol mobilization. In contrast, 2-AG was unable to abolish the developmental arrest induced by cholesterol depletion in *sbp-1(ep79)* animals (Fig 5B). This suggests that 2-AG promotes mobilization of cholesterol through a pathway that requires the transcriptional activity of SBP-1/SREBP. Consistent with this result, 2-AG was unable to suppress dauer formation in *ncr-2; ncr-1* mutants exposed to *sbp-1* RNAi, even though this Daf-c phenotype was largely suppressed by raising the cholesterol concentration in the growth media (Fig 5C). Surprisingly, this high cholesterol concentration did not rescue the Daf-c phenotype of *ncr-2; ncr-1* mutants subjected to *sbp-1* RNAi. This suggests that *sbp-1* genetically interacts with the Niemann Pick proteins to regulate intracellular cholesterol trafficking. These findings agree with previous studies showing that *ncr-1* is transcriptionally regulated by SBP-1 [37] and that mammalian NPC-1 expression is sterol-controlled by SREBP [38].

Finally, we found that *sbp-1* animals displayed elevated 2-AG levels compared with control animals (Fig 5D). This could be due to a compensatory mechanism in order to optimize cholesterol trafficking in animals depleted of SBP-1.

Taken together, our results suggest that SBP-1, in concert with the Niemann-Pick homologs, plays an important role in the 2-AG signal transduction pathway to mobilize cholesterol.

### Mobilization of sterols by 2-AG is controlled by the insulin pathway

The DAF-2/IIS and DAF-7/TGF-β signaling comprise the major endocrine pathways modulating the conversion of cholesterol into DA [13]. In a previous study it was shown that temperature-sensitive *daf-7* mutants are hypersensitive to cholesterol depletion and form dauer larvae in the absence of external cholesterol already at 20°C [14]. More recently, we reported that upon cholesterol deprivation 2-AG partially rescues the dauer arrest of *daf-7* animals [15]. We determined that 2-AG also prevents dauer formation in mutants which are defective in core components of the DAF-7/TGF-β signaling pathway (S5A Fig), suggesting that 2-AG functions independently of this pathway.

**Fig 6:**
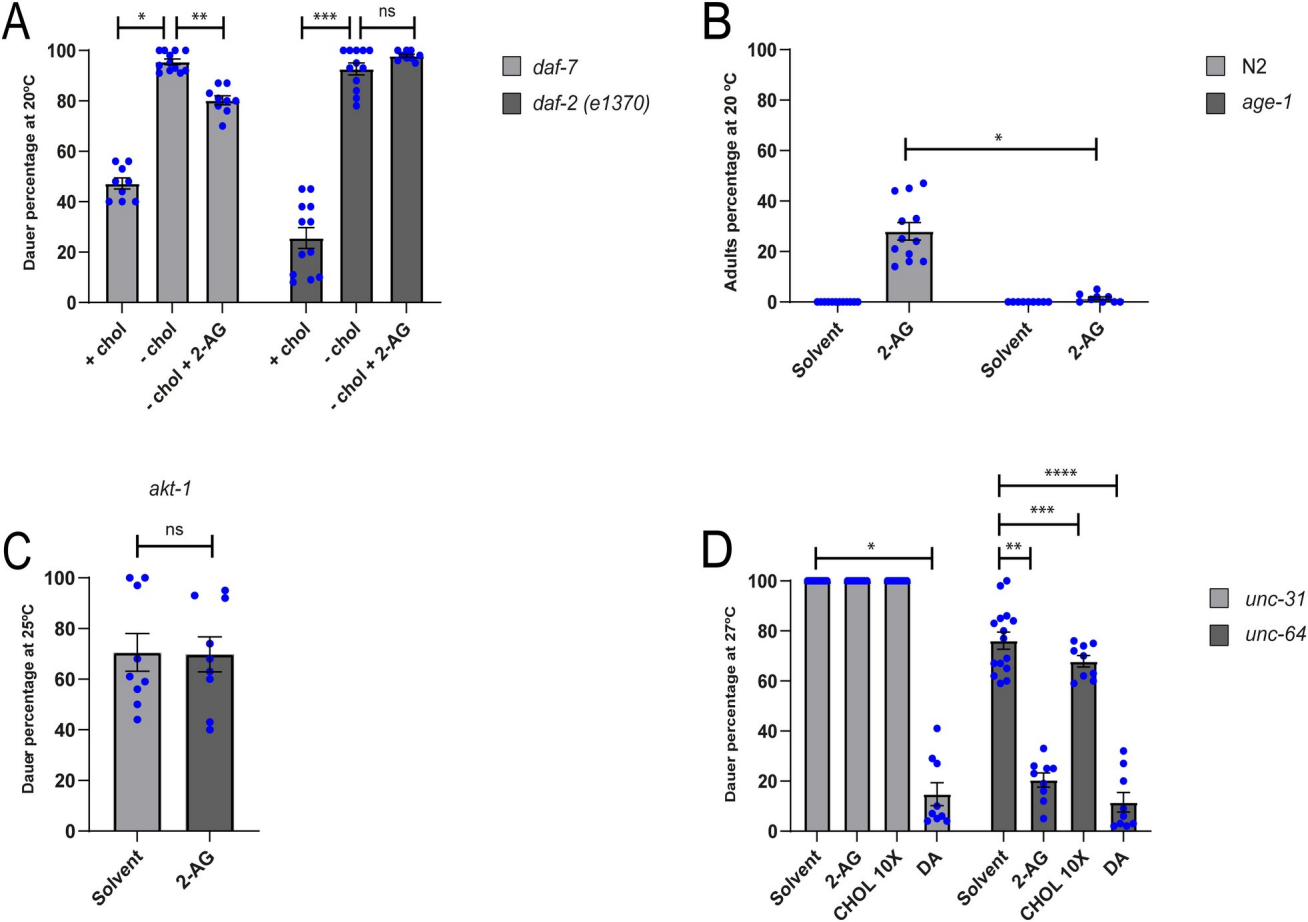
DAF-2 and UNC-31 are required for 2-AG-dependent cholesterol mobilization. (A) *daf-7* and *daf-2(e1370*) were grown at 20°C either in cholesterol or in a sterol-free media during one generation. When indicated the media was supplemented with either 13 μM cholesterol or 50 μM 2-AG. All Pairwise are Multiple Comparison Procedures (Dunn’s Method), *p < 0.05. **p < 0.05. ***p < 0.05. All values are from n ≥ 3 independent experiments shown as Mean ± SEM. ns = not significant. (B) N2 and *age-1* were grown for two generations in media with 0 μg/ml cholesterol at 20°C. Mann-Whitney rank sum test, *p < 0.001. All values are from n ≥ 3 independent experiments shown as Mean ± SEM. (C) *akt-1* was grown in media with 0 μg/ml cholesterol during one generation at 25°C. All values are from n = 3 independent experiments shown as Mean ± SEM. ns = not significant. (D) 2-AG is unable rescue the daf-c phenotype of *unc-31* grown at 27°C. All Pairwise are Multiple Comparison Procedures (Dunn’s Method), *p < 0.05. All Pairwise are Multiple Comparison Procedures (Holm-Sidak method), **p < 0.05. ***p < 0.05. ****p < 0.05. All values are from n ≥ 3 independent experiments shown as Mean ± SEM. N2 is the *C*. *elegans* wild-type strain.

Interestingly, *daf-2(e-1370)* mutants with reduced Insulin-IGF-1 receptor signaling also form about 90% dauers at 20°C in the first generation in the absence of cholesterol (Fig 6A). Nevertheless, unlike *daf-7* mutants, 2-AG could not suppress the dauer arrest of *daf-2* animals starved from cholesterol at 20°C (Fig 6A) or at 23°C (S5B Fig). This suggests that 2-AG-mediated mobilization of cholesterol depends on the IIS pathway. We also tested the requirement of phosphoinositide-3 kinase AGE-1/PI3K and of the serine threonine kinase AKT-1 that act downstream of the DAF-2 insulin receptor. 2-AG was unable to suppress the dauer-like phenotype of both single mutants growing under sterol depleted conditions (Fig 6B and 6C).

**Fig 7:**
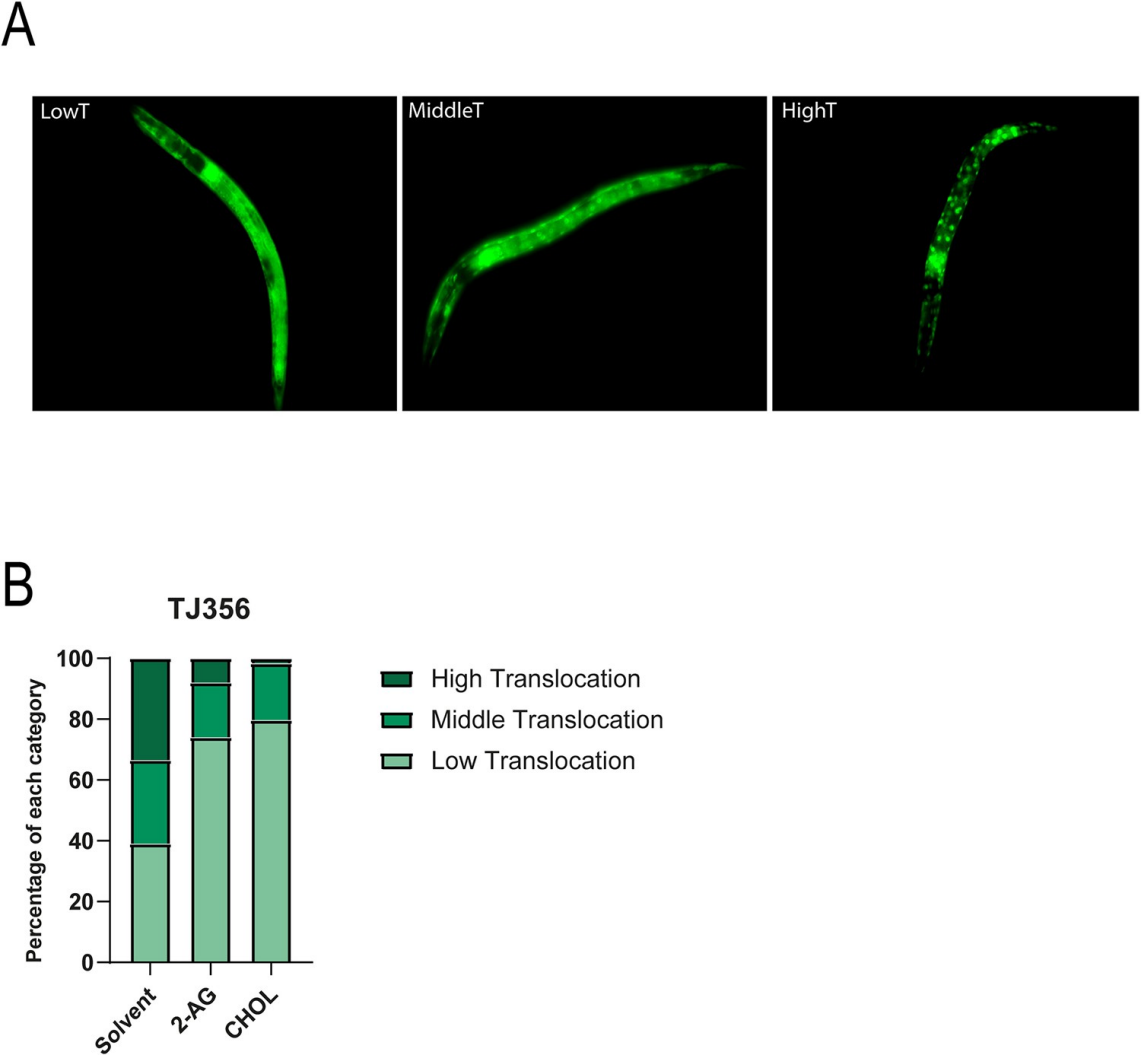
2-AG signaling inhibits nuclear translocation of DAF-16 induced by cholesterol depletion. (A) DAF-16::GFP nuclear translocation group classification. Translocation categories were placed into 3 classes as shown in the picture. Animals which exhibited DAF-16::GFP nuclear accumulation between 0–10% of total DAF-16::GFP were classified as Low Translocation (LowT), 30–50% as Middle Translocation (MiddleT) and 70–90%, High Translocation (HighT). All animals shown are in a L2 larva stage. (B) Nuclear translocation of DAF-16::GFP in worms grown under normal cholesterol concentration (CHOL) and after cholesterol depletion for two generations in the absence (solvent) or presence of 2-AG. 69 individual worms were analyzed for the solvent condition, 66 for 2-AG and 64 for CHOL.

The DAF-2/IIS pathway has several physiological functions. Thus, it has been suggested that it is involved in the process of dauer formation by regulating the levels of DA via DAF-16 [9]. Indeed, previous work showed that the substitution of dietary cholesterol by lophanol, a sterol from which DA cannot be derived [9,39], leads to translocation of DAF-16 to the nucleus [9]. In agreement with this report [9] we found that after two generations of cholesterol depletion, a DAF-16::GFP fusion protein exhibited significantly higher accumulation in the nucleus compared to worms grown on cholesterol supplemented media (Fig 7). Exogenous 2-AG largely inhibited the nuclear localization of DAF-16 (Fig 7), adding important evidence to the hypothesis that the eCBs stimulate the mobilization of cholesterol and its conversion into DA.

Finally, we found that the 2-AG antagonist, NMP331, does not enhance the Daf-c phenotype at 20°C of *daf-2(e1370*) mutants (S5C Fig). This result shows that NMP331 requires a functional IIS signaling pathway for its effect on cholesterol trafficking. Taken together, our results suggest that IIS and 2-AG converge in a process essential for cholesterol mobilization.

### UNC-31 and HID-1 are required for 2-AG-dependent cholesterol mobilization

We next sought to determine whether 2-AG rescued dauer formation in worms deficient in proteins that act upstream DAF-2, such as UNC-64/syntaxin [40] and UNC-31/CAPS [41]. *unc-64* and *unc-31* mutants have constitutive dauer formation at 27°C but not at 25°C (Fig 6D) [42]. Dauer formation of *unc-64* mutant animals was markedly suppressed at 27°C by either 2-AG or DA under normally dietary cholesterol. As expected, high concentrations of cholesterol also suppressed dauer formation of *unc-64* mutants at 27°C (Fig 6D). In contrast, 2-AG was unable to suppress the Daf-c phenotype of *unc-31* mutants, suggesting that its gene product is essential for the 2-AG-dependent mobilization of cholesterol (Fig 6D). Consistent with a role of UNC-31 in cholesterol homeostasis, we found that DA rescues the dauer arrest of *unc-31* animals (Fig 6D). *unc-31* encodes the *C*. *elegans* homolog of mammalian Ca^2+^ activated protein for secretion (CAPS), required for the regulated release of dense core vesicles (DCVs), which contain biogenic amines, neuropeptide, and insulins [41,43–45]. In addition, 2-AG was unable to rescue the Daf-c phenotype at 27°C of animals deficient in HID-1, a key component in the secretion of DCVs [46] (S6A Fig). HID-1 is expressed in all neuron and gut cells of *C*. *elegans*. Expression of HID-1 under the *rab-3* promoter in neurons in a *hid-1(sa722)* mutant background produced a slight, but statistically significant, rescue of the effect of 2-AG on dauer formation (S6B Fig). In contrast, 2-AG failed to rescue dauer formation when HID-1 was expressed under the *ges-1* promoter in the gut of *hid-1* animals (S6B Fig). Taken together, these experiments indicate that diminished neural release of DCVs impairs the effect of 2-AG on cholesterol mobilization.

## Discussion

Although cannabis extracts have been used in folklore medicine for centuries, the past few years have increased the interest in the medicinal use of cannabinoids, the bioactive components of the cannabis plant, in the treatment of many diseases of the nervous system. We have recently demonstrated that 2-AG and AEA, the best characterized eCBs reverse the blockade of intracellular trafficking of cholesterol in *C*. *elegans* [15]. Since the endogenous cannabinoid 2-AG and AEA are synthesized within the brain and CNS [47], unraveling the molecular basis of cholesterol mobilization by eCBs in *C*. *elegans* could have important implications for a greater understanding of human pathological conditions associated with impaired cholesterol homeostasis.

Cannabinoids primarily activate G_αo_-coupled cannabinoid receptors 1 and 2 (CB1 and CB2) [20,22]. CB1 is localized primarily in the brain and CNS, whereas CB2 is restricted to the periphery and certain leukocytes [48]. Although initial reports suggested that nematodes lacked a canonical CB receptor [49,50], it has been determined that *C*. *elegans* also possesses cannabinoid-like receptors [17,18]. NPR-19 is a functional orthologue to the mammalian CB1/2, and NPR-32, a functional orthologue to GPR18 and GPR55 [17]. Here we show that the modulation of cholesterol homeostasis by 2-AG is independent of the GPCRs NPR-19 and NPR-32 signaling. Instead, we found that 2-AG displays interactions with components of the insulin/IGF1 signaling, a major endocrine pathway modulating DA production and dauer formation. We determined that reduction of the IIS pathway by mutations in the insulin/IGF receptor homolog *daf-2*, the phosphoinositide-3 kinase AGE-1/PI3K or the serine threonine kinase AKT-1 result in animals that are not rescued by 2-AG in a cholesterol depleted medium (Fig 6). Since reduction of DAF-2/IIS pathway activity significantly affects 2-AG signaling, we asked whether insulin like peptide secretion controls eCB-mediated cholesterol homeostasis. We found that the calcium-activated regulator of dense-core vesicle release (DCVs), UNC-31/CAPS, which functions in the nervous system to mediate release of insulin like peptides [41,43–45] is essential for 2-AG-mediated stimulation of cholesterol mobilization (Fig 6D). It is puzzling that *unc-64* does not behave in the same way that *unc-31* (Fig 6D) as these two genes are involved in mediating Ca^2+^ regulated neurotransmitter secretion [42,51]. Nevertheless, we cannot exclude a role for UNC-64/syntaxin in 2-AG-mediated cholesterol homeostasis since in our experiments we used *unc-64 (e246)* which is a partial loss of function allele [42], while *unc-31 (e928)* is a deletion that removes most of the *unc-31* gene [42].

In summary, the essentiality of *unc-31* combined with the requirement of *hid-1* for 2-AG signaling, suggests that neural release of DCVs is required for 2-AG regulation of cholesterol homeostasis. We hypothesize that 2-AG stimulates cholesterol mobilization through the release of insulin-like peptides.

**Fig 8:**
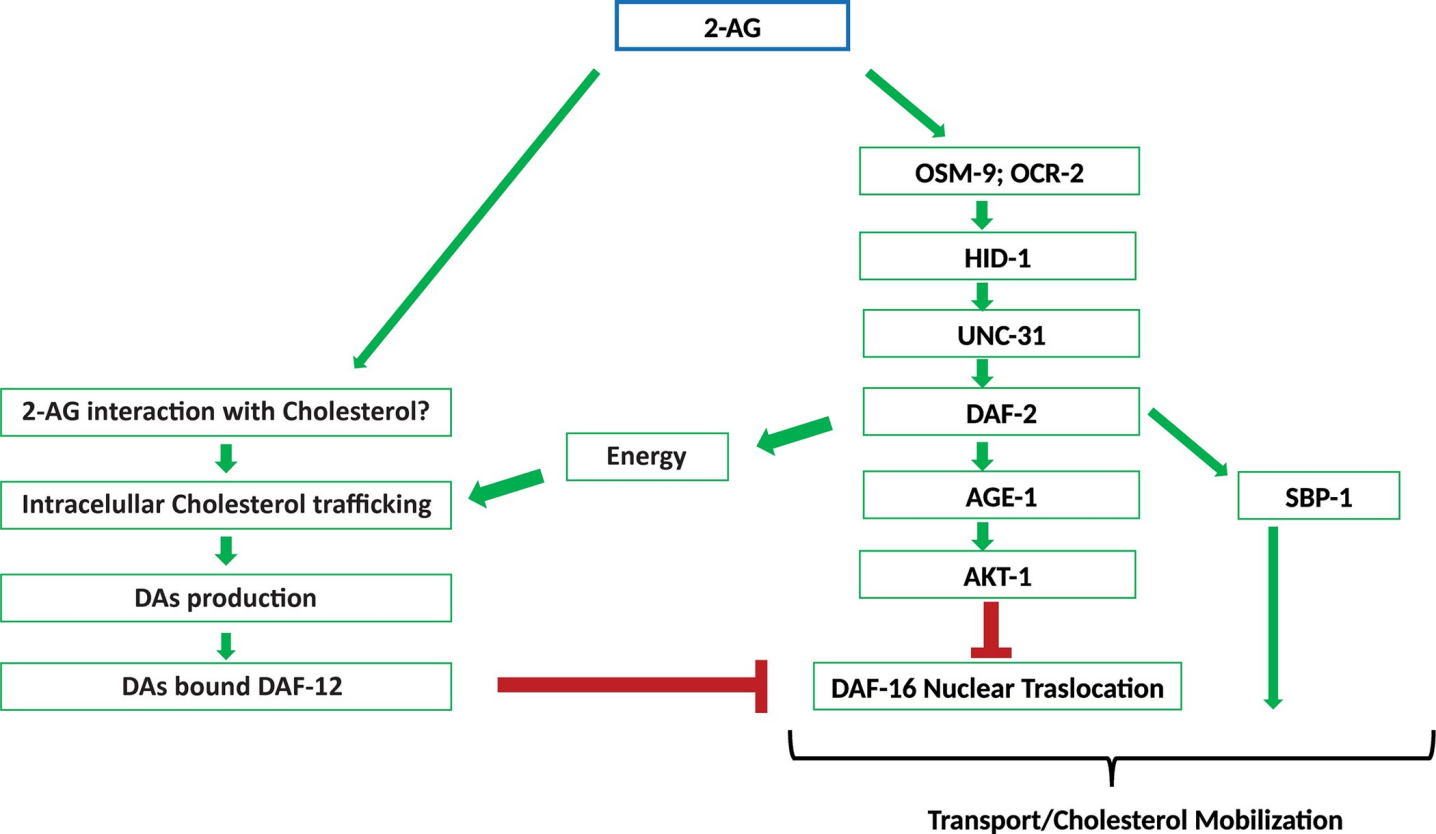
Working model of 2-AG dependent mobilization of cholesterol. Under interaction of 2-AG with an unknown target in sensory neurons, multiple signaling mechanisms are likely to converge on activation of the IIS pathway and inhibition of DAF-16/FOXO. Signaling through the DAF-2 insulin receptor has several functions and could be involved in modulation of the intracellular traffic of cholesterol. The interaction of SBP-1 with the IIS pathway remains to be established, but is likely that such interaction is essential for 2-AG-dependent mobilization of cholesterol. For more details see the text.

The simplest model consistent with previous [15] and present results is that 2-AG has a dual role, promoting the release of insulin peptides contained in DCVs and removing cholesterol from internal pools (Fig 8). It is not known how and where worms store the internal sterol pools. Even though cholesterol is associated with cell membranes and interacts with multiple lipid species, very little is known about how lipids influence cholesterol trafficking. One of the few examples is the positive effect of the phospholipid lysobisphosphatidic acid on the trafficking of cholesterol through the endolysosomal compartment [52]. Owing to the huge diversity of membrane lipids, multiple other lipid species might emerge as additional modulators of the cholesterol trafficking process. eCBs are amphiphilic molecules derived from phospholipids that are unlikely to diffuse passively in the membrane. Several reports have shown that cholesterol behaves as a specific binding partner for eCBs [53,54]. Following an initial interaction of either 2-AG or AEA with cholesterol, mediated by the establishment of hydrogen bonds, they are attracted towards the membrane interior forming a molecular complex [53,54]. This raises the possibility that the interaction of 2-AG with cholesterol enhances the intracellular trafficking of sterols to steroidogenic tissues, positively affecting production of DAs. This regulated transport of cholesterol demands energy [55]. As upregulation of the insulin pathway is linked to increased metabolic rates [56], 2-AG-mediated activation of the DAF-2 pathway may induce a metabolic shift to provide the fuel needed to meet the high energy demands of *C*. *elegans* cholesterol mobilization (Fig 8). In addition, stimulation of the IIS pathway should enforce, together with DA-bound DAF-12, the DAF-16/FOXO cytoplasmic retention to rescue the arrest of cholesterol depleted worms (Fig 8).

Many of the physiological effects produced by eCBs are not completely understood. As we report here, some of them may reflect their influence on cholesterol trafficking. Interestingly, eCBs and eCB agonists are known to increase the hepatic expression of SREBP in mice [57]. Here we show that the simple orthologue of mammalian SREBP in *C*. *elegans*, SBP-1, plays an important role in 2-AG mobilization of cholesterol (Fig 5B). Notably, RNAi of SBP-1 increased the penetrance of Daf-c defects seen in *daf-2 (e1370*) when grown in normal dietary cholesterol (S7 Fig). Although experimental evidence has demonstrated that *daf-2* controls the expression of numerous genes predicted to participate in fatty acid metabolism [58], it is not clear how DAF-2 and its regulatory targets regulates lipid metabolism. Our findings indicate that SBP-1 and IIS converge on a critical physiological process potentially related to the role of eCBs in cholesterol availability (Fig 8). Future work should elucidate the nature of such interaction.

The specific molecular mechanisms through which *C*. *elegans* sensory neurons detect eCBs remain to be deciphered, however our findings suggest a key role for the *osm-9* and *ocr-2* TRPV genes in the control of cholesterol trafficking mediated by 2-AG. We found that both *osm-9* and *ocr-2* arrest already in the first generation without externally provided sterols (Fig 3), reminiscent of *fat-3* and *fat-4* mutants which are aberrant in cholesterol mobilization because they are unable to synthesize AEA and 2-AG [15]. Moreover, the arrest of *osm-9* and *ocr-2* mutants is not rescued by 2-AG, strongly suggesting that OSM-9/OCR-2 TRPV channel is essential for 2-AG-dependent cholesterol mobilization. Since the TRPV family encodes for non-selective cation channels with a preference for Ca^2+^ [30], it is possible that OCR-2/OSM-9 may directly activate insulin secretion trough UNC-31, a Ca^2+^ dependent regulator of DCVs release.

Although the exact mode of activation of OSM-9 and OCR-2 in neurons is partly understood, our data are consistent with the hypothesis that 2-AG acts upstream of the TRPV channel. Interestingly, it has been suggested that G-protein-coupled lipid signaling pathways regulate TRPV channel signaling in chemosensory neurons [59]. This yet to be identified pathway could be specifically inhibited by the cannabinoid based calcium blocker NMP331, that potently competes with 2-AG in cholesterol mobilization (S4A Fig).

Our insights in the role of eCBs in nematode cholesterol homeostasis has added an important new piece of information. Yet, the puzzle remains incomplete, and many mechanistic questions have yet to be answered. It seems plausible that both worms and mammals possess a fully functional eCB signaling pathway that regulates cholesterol homeostasis. Further dissecting cannabinoid regulation of lipid homeostasis in the context of larger endocrine networks should reveal how these processes alter disease states, health and possibly longevity.

## Materials and methods

### Materials

2-AG and 2-AGE were purchased from Cayman Chemical (Ann Arbor, Michigan, USA) and stock solutions were made in acetonitrile at 1 mg/ml and are stored at -80°C. Cholesterol, Dubelcco´s medium (DMEM) and antioxidant BHT were purchased from Sigma (Sigma-Aldrich, St. Louis, Missouri, USA). Δ4-DA and Δ7-DA were provided by Prof. H.-J. Knölker. All buffers, salts and chemical were reagent grade and were used without further treatments. Unless specified, all reagents were purchased from Merck or Sigma. The cannabinoid receptor ligands (NMP compounds) 241, 242, and 243 are named as compounds 40, 54 and 41 in reference 32. Compound NMP331 is named as compound 10 in reference 33.

### Nematode maintenance and strains

Standard *C*. *elegans* culture and molecular biology methods were used. Strains were cultured at 15°C or 20°C on nematode growth media (NGM) agar plates with the *E*. *coli* OP 50 strain as a food source [60]. The wild type strain was Bristol N2. Some strains were provided by the *Caenorhabditis* Genetics Center (CGC) at the University of Minnesota. The strains *hid-1 (sa722)*, *hid-1 (sa722); lin-15 (n765); jsEx897 [rab-3p-HID-1-GFP]*, *hid-1 (sa722); lin-15 (n765); jsEx909 [ges-1p-HID-1-GFP]*, *hid-1 (sa722); lin-15 (n765); jsEx896 [hid-1p-HID-1-GFP] were* kindly provided by D. Rayes. The strains used were: N2 Bristol (wild-type), *daf-7(e1372)*, *daf-2(e1370)*, *daf-2(e1368)*, *fat-4 (ok958)*, *ncr-1 (nr2022); ncr-2 (nr2023)*, *npr-19 (ok2068)*, *tyra-3(ok325)*, *ckr-2(tm3082)*, *gar-2(ok520)*, *gar-3(vu78)*, *gar-1(ok755)*, *ser-2(ok2103)*, *npr-11 (ok594)*, *ser-1(ok345)*, *ser-5(ok3087)*, *npr-5(ok1583)*, *dop-1(vs101)*, *mod-1(ok103)*, *octr-1(ok371)*, *npr-16(ok1541)*, *ser-7(tm1325)*, *ser-4(ok512)*, *dop-2(vs105)*, *npr-24(ok3192)*, *dop-1(ok398)*, *npr-32(ok2541)*, *npr-35 (ok3258)*, *gnrr-1 (ok238)*, *age-1 (hx546)*, *akt-1 (ok525)*, *daf-4* (e1364), *daf-8* (e1393), *unc-64 (e246)*, *unc-31(e928)*, *daf-14 (m77)*, *faah-4 (lt121)*, *sbp-1 (ep79)*, *nhr-8 (ok186)*, *ocr-1 (ok132)*, *ocr-2 (ok1711)*, *ocr-3 (ok1559)*, *ocr-4 (vs137)*, *osm-9 (ok1677)*, *tph-1 (mg280)*, *cca-1 (ok3442*), *zIs356 [daf-16p*::*daf-16a/b*::*GFP + rol-6(su1006)]*. *hid-1 (sa722)*, *hid-1 (sa722); lin-15 (n765); jsEx897 [rab-3p-HID-1-GFP]*, *hid-1 (sa722); lin-15 (n765); jsEx909 [ges-1p-HID-1-GFP]*, *hid-1 (sa722); lin-15 (n765); jsEx896 [hid-1p-HID-1-GFP]; xuEx1978 [Psra-6*::*Gcamp6(f)*, *Psra-6*::*DsRed]*.

### Preparation of sterol-depleted plates and sterol-deprived worm culture

To obtain sterol-free conditions, agar was replaced by ultrapure agarose and peptone was omitted from plates as described earlier [15]. Briefly, agarose was washed three times overnight with chloroform to deplete the trace sterols in it. Salt composition was kept identical to NGM plates. As a food source, *E*. *coli* NA22 grown overnight in sterol-free culture medium DMEM was used. Bacteria were rinsed with M9 buffer and 20 times concentrated. Bleached embryos were grown for one generation on sterol-free agarose plates. The resulting gravid adults were bleached and the obtained embryos were used in various assays.

The animals grown for two generations in the absence of cholesterol arrest as L1-L2* larvae [9]. The bypass of the L2* larval arrest is indicated as % of developed adults.

### Generation of *daf-7; npr-19* and *npr-19; npr-32* double mutants

The *daf-7* and *npr-32* mutants were each crossed into *npr-19* to generate double mutants by standard methods. Crosses were confirmed by PCR genotyping and constitutive dauer arrest at 25°C.

### RNAi screen

To identify genes controlling cholesterol trafficking by 2-AG we carried out RNAi enhancer screen of *daf-7 (e1372)* mutants, which have partially diminished internal pool mobilization of cholesterol [14]. The RNAi mini-library was generated by selecting 16 clones from the Ahringer RNAi library. RNAi screen was carried out on plates supplemented with cholesterol (13 μM final concentration). The enhanced effect of RNAi was examined in the F1 generation. To this end bleached embryos were left to hatch overnight in M9 at 20°C and the resulting L1s were seeded to RNAi plates and grown at 20°C. First, we surveyed for enhancement of the Daf-c phenotype (S2 Table) and, in a secondary survey, screened for loss of 2-AG-dependent rescue of the developmental arrest caused by two generations of cholesterol depletion.

### Dauer formation assays

Dauer assays were performed as previously described [15]. In general, 60–80 L1s or embryos were transferred to NGM plates seeded with *E*. *coli* (HT115, NA22 or OP50). eCBs (final concentration 50 μM) or NMP331 (final concentration 1 μM or as indicated in Fig 4) were added to the bacteria immediately prior to seeding. The final concentrations of these compounds were calculated according to the volume of the NGM agar used for the preparation of the plates. Depending on the sort of essays after 3, 4 or 5 days the dauer percentage was scored. Δ7-DA was used alternatively in dauer rescue experiments at a concentration of 90nM.

### Lipid Extraction and endocannabinoid analysis by HPLC-MS/MS

The protocol was adapted from Folch (1957) [61]. Briefly, lipid extracts were made from approximately 200 mg of frozen worm pellets grown at 20°C. Pellets were washed with M9 buffer, then re-suspended in 1.3 ml pure methanol and sonicated three times for 30 seconds. After sonication, 2.6 ml of chloroform and 1.3 ml 0.5 M KCl/0.08 M H3PO4 were added. Butylated hydroxytoluene (BHT, 0.005% v/v) was added to prevent lipid oxidation. Samples were then sonicated for 15 min, vortexed twice for 1 min and centrifuged for 10 min at 2.000 x g to induce phase separation. The lower, hydrophobic phase was collected, dried under constant nitrogen stream, re-suspended in 100 μl of acetonitrile and loaded into dark caramel tubes using a glass pipette.

2-AG was quantified from nematode samples by liquid chromatography (Ultimate 3000 RSLC Dionex, Thermo Scientific) coupled with an ESI triple quadrupole mass spectrometer (TSQ Quantum Access Max (QQQ), Thermo-Scientific) as previously described [15].

### DAF-16:GFP expression analysis

A stably integrated DAF-16::GFP, TJ356 was used. The expression of GFP was observed using a Nikon Eclipse 800 microscope equipped with a fluorescent light source. The images were captured with a Andor Clara digital camera.

To observe GFP distribution, 15–25 animals were mounted on an agar pad containing 20 mM sodium azide and analyzed immediately. Animals on the pad were scored for a maximum of 10 min. DAF-16::GFP distribution was categorized based on the accumulation of GFP in the nucleus or the cytoplasm (diffuse form) in the whole animal. Individual animals were classified based on the presence of nuclear DAF-16::GFP in approximately 90%, 70% (High translocation), 50%, 30% (Middle traslocation), 10% or none (Low Translocation) of the body cells, as shown in S7 Fig. All scored animals were at the L2 stage after 72 hs of growing in the second generation under free sterol conditions.

### Two-electrode voltage clamp recordings in *Xenopus oocytes*

For electrophysiological recording in *Xenopus levis* oocytes, OSM-9 and OCR-2 subunits subcloned into a modified pGEMHE vector were used. cRNAs were in vitro transcribed from linearized plasmid DNA templates using Large Scale RNA Production System (Promega, Madison, WI, USA). Xenopus oocytes were injected with 50 nl of RNase-free water containing 1.0 ng of cRNA (at a 1:1 molar ratio for heteromeric receptors) and maintained in Barth’s solution [in mM: NaCl 88, Ca(NO3_)2_ 0.33, CaCl_2_ 0.41, KCl 1, MgSO_4_ 0.82, NaHCO_3_ 2.4, HEPES 10] at 18°C. Electrophysiological recordings were performed at −60 mV under two-electrode voltage-clamp, with an Oocyte Clamp OC-725B or C amplifier (Warner Instruments Corporation, Hamden, CT, USA). Recordings were filtered at a corner frequency of 10 Hz using a 900 BT Tunable Active Filter (Frequency Devices Inc., Ottawa, IL, USA). Data acquisition was performed using a Patch Panel PP-50 LAB/1 interphase (Warner Instruments Corp., Hamden, CT, USA) at a rate of 10 points per second. Both voltage and current electrodes were filled with 3M KCl and had resistances of ∼1MΩ. Data were analysed using Clampfit from the pClamp 6.1 software (Molecular Devices, Sunnyvale, CA). During electrophysiological recordings 100 μM 2-AG was added to the perfusion solution. Recording was performed at room temperature and heat-stimulation (~ 36°C) by perfusion of heated Barth’s. The temperature of perfused bath solutions was checked with a TC-344B temperature controller (Warner Instruments) located near the oocytes. Mean ± SEM of current amplitudes of responses to temperature, 100 μM 2-AG and temperature plus 100 μM 2-AG in oocytes injected with either OSM-9, OCR-2 or OSM-9/OCR-2 were calculated using Prism 6 software (GraphPad Software Inc., La Jolla, CA, USA).

### ASH calcium imaging

Animals expressing GCaMP6 in the ASH (xuEx1978 [Psra-6::Gcamp6(f), Psra-6::DsRed]) were immobilized in a PDMS microfluidic olfactory chip [62,63] and exposed to either a control buffer or a stimulus buffer containing 100mM 2-AG (Cayman Chemical Co. #62160). The required volume of 2-AG required to make 100mM solution was dissolved in 0.1% ethanol before being added to S-Basal buffer. Since the 2-AG stock solution was dissolved in acetonitrile, we added an equivalent volume of acetonitrile and 0.1% ethanol S-basal to make the control buffer. This ensured that responses were specific to 2-AG. The stimulus protocol was based on previously described exposure experiments [59]. Animals were allowed to stabilize in the olfactory chip for at least 5 minutes before recording. Recording was performed at 10x magnification on an AxioObserver A1 inverted microscope (Zeiss) connected to a Sola SE Light Engine (Lumencor) and an ORCA-Flash 4.0 digital CMOS camera (Hamamatsu). Micromanager Software [64] was used to control image acquisition. Recording was performed at 10 frames per second and 4x4 image binning. An Arduino was used to control pinch valves to direct stimulus and control buffer to the nose of animals in the following sequence: 6 second baseline, 4 second stimulation, 10 second interstimulus interval, 4 second stimulation, 6 second washout. GCaMP fluorescence was extracted using Matlab (Mathworks) scripts and the results were plotted using GraphPad Prism.

## Supporting information

S1 Fig TPH-1 is not required for 2-AG-dependent cholesterol mobilization. (A) N2 and *tph-1* were grown for two generations in media with 0 μg/ml cholesterol at 20°C. Mann-Whitney rank sum test, *p < 0.001. t-test, **p < 0.001. All values are from n = 3 independent experiments shown as Mean ± SEM. N2 is the *C*. *elegans* wild-type strain.
(TIF)

S2 Fig 2-AG did not elicit currents in Xenopus oocytes expressing OSM-9 and OCR-2 channels. (A) Representative traces of responses (above) and temperature (below) in *Xenopus oocytes* injected with cRNAs encoding OSM-9, OCR-2, OSM-9/OCR-2. (B) Mean ± SEM of heat evoked currents. Amplitudes were calculated by measuring the differences between the peak inward currents and baseline marked with dotted lines (***p = 0.0001, n ≥ 5 per group, ANOVA followed by a Bonferroni’s multi-comparison test). (C) Representative traces of responses to 100 μM 2-AG either at room temperature (grey traces) or during a temperature ramp in oocytes expressing OSM-9, OCR-2 or OSM-9/OCR-2. (D) Mean ± SEM of current amplitudes of responses to temperature, 100 μM 2-AG and temperature plus 100 μM 2-AG in oocytes injected with either OSM-9, OCR-2 or OSM-9/OCR-2 (**** p < 0.0001, n ≥ 3 oocytes per group, two-way ANOVA followed by a Bonferroni multi- comparison test).
(TIF)

S3 Fig ASH calcium levels are not affected by 2-AG. Calcium imaging traces of animals expressing GCaMP6 in the ASH (xuEx1978 [Psra-6::Gcamp6(f), Psra-6::DsRed]) during exposure to 2-AG. Average responses for all animals (A) are indicated by the dark blue line and the shaded area represents the SEM. The same results are shown as individual traces for each animal (B). The dark grey bars indicate 4 second exposure to buffer containing 100 mM 2-AG.
(TIF)

S4 Fig NMP331 enhances dauer formation in *daf-7* mutants which can be overcome by 2-AG supplementation and induces dauer-like arrest in *npr* mutants starved for sterols. (A) 2-AG antagonizes the effect of 1 μM of NMP331 in a *daf-7* worm at 20°C. All pairwise are multiple comparison procedures (Holm-Sidak method), *p < 0.001. All values are from n = 3 independent experiments shown as Mean ± SEM. (B) N2 and *npr* animals undergo a dauer-like arrest induced by NMP331 in the first generation when grown in cholesterol free medium. The concentration of NMP331 was 50 μM. ns = not significant (C) NMP331 enhances dauer formation in *daf-7; npr-19* animals in media with cholesterol 13 μM. Mann-Whitney rank sum test, *p < 0.001. All values are from n = 3 independent experiments are shown as Mean ± SEM.
(TIF)

S5 Fig Mobilization of cholesterol by 2-AG is independent of the *daf-7* pathway. (A). *Daf-4* was grown at 20°C while *daf-14* and *daf-8* were grown at 25°C under normal dietary cholesterol (13 μM). t-test, *p < 0.001. **p < 0.001. ***p < 0.002. All values are from n = 3 independent experiments shown as Mean ± SEM. (B) *daf-7* and *daf-2* were grown at 23°C in a sterol-free media during one generation. t-test, *p < 0.001. All values are from n ≥ 3 independent experiments shown as Mean ± SEM. ns = not significant. (C) NMP331 does not enhance the daf-c phenotype of *daf-2* mutants. Mann-Whitney rank sum test, *p < 0.001. All values are from n ≥ 3 independent experiments shown as Mean ± SEM. ns = not significant.
(TIF)

S6 Fig Mobilization of cholesterol by 2-AG is dependent of *hid-1*. (A) *Hid-1* is required for 2-AG dependent mobilization of cholesterol. Worms were grown at 27°C under normal dietary cholesterol. All pairwise are multiple comparison procedures (Holm-Sidak method), *p < 0.05. All values are from n = 3 independent experiments shown as Mean ± SEM. ns = not significant. (B) HID-1 expression in neurons in a *hid-1* background restores the 2-AG-dependent mobilization of cholesterol. Worms were grown at 27°C under normal dietary cholesterol. Mann-Whitney rank sum test, *p < 0.001. All values are from n ≥ 3 independent experiments shown as Mean ± SEM. ns = not significant.
(TIF)

S7 Fig s*bp-1* RNAi increases the *daf-c* phenotype of *daf-2*. *daf-2* was grown in 13 μM cholesterol at 20°C. Mann-Whitney rank sum test, *p < 0.005. All values are from n = 3 independent experiments shown as Mean ± SEM.
(TIF)

S1 Table Adult percentage in orthologues of CB1/2 mutants grown for two generations in the absence of cholesterol arrest as L2* larvae. (DOCX)

S2 Table Dauer percentage in RNAi enhancer screen essays on *daf-7 (e1372)* worms. For details see text.
(DOCX)

## References

[1] Kritchevsky SB, Kritchevsky D. Serum cholesterol and cancer risk: An Epidemiologic Perspective. Annu Rev Nutr. 1992. doi: 10.1146/annurev.nu.12.070192.002135 1503812

[2] Woollett LA. Where does fetal and embryonic cholesterol originate and what does it do? Annual Review of Nutrition. 2008. pp. 97–114. doi: 10.1146/annurev.nutr.26.061505.111311 18662139

[3] Cai J, Pajak A, Li Y, Shestov D, Davis CE, Rywik S, et al. Total cholesterol and mortality in China, Poland, Russia, and the US. Ann Epidemiol. 2004;14: 399–408. doi: 10.1016/j.annepidem.2003.10.012 15246328

[4] Iqbal J, Hussain MM. Intestinal lipid absorption. Am J Physiol Endo-crinol Metab. 2009;296: 1183–1194. doi: 10.1152/ajpendo.90899.2008 19158321 PMC2692399

[5] Martins IJ, Hone E, Foster JK, Sünram-Lea SI, Gnjec A, Fuller SJ, et al. Apolipoprotein E, cholesterol metabolism, diabetes, and the convergence of risk factors for Alzheimer’s disease and cardiovascular disease. Molecular Psychiatry. 2006. pp. 721–736. doi: 10.1038/sj.mp.4001854 16786033

[6] Patel TN, Shishehbor MH, Bhatt DL. A review of high-dose statin therapy: targeting cholesterol and inflammation in atherosclerosis. Eur Heart J. 2007;28: 664–672. doi: 10.1093/eurheartj/ehl445 17242008

[7] Paul H A Steegmans, Durk Fekkes, Arno WHoes, Annette AA Bak, Emiel van der Does DEG. Low serum cholesterol concentration and serotonin metabolism in men. BMJ. 1996;312: 221. doi: 10.1136/bmj.312.7025.221 8563588 PMC2350030

[8] Sturley SL, Patterson MC, Balch W, Liscum L. The pathophysiology and mechanisms of NP-C disease. Biochim Biophys Acta. 2004;1685: 83–7. doi: 10.1016/j.bbalip.2004.08.014 15465429

[9] Matyash V, Entchev E V, Mende F, Wilsch-Brauninger M, Thiele C, Schmidt AW, et al. Sterol-Derived Hormone(s) Controls Entry into Diapause in Caenorhabditis elegans by Consecutive Activation of DAF-12 and DAF-16. PLoS Biol. 2004;2: 1561–1571. doi: 10.1371/journal.pbio.0020280 15383841 PMC517820

[10] Matyash V, Geier C, Henske A, Mukherjee S, Hirsh D, Thiele C, et al. Distribution and transport of cholesterol in Caenorhabditis elegans. Mol Biol Cell. 2001;12: 1725–1736. doi: 10.1091/mbc.12.6.1725 11408580 PMC37336

[11] Motola DL, Cummins CL, Rottiers V, Sharma KK, Li T, Li Y, et al. Identification of Ligands for DAF-12 that Govern Dauer Formation and Reproduction in C. elegans. Cell. 2006;124: 1209–1223. doi: 10.1016/j.cell.2006.01.037 16529801

[12] Wollam J, Antebi A. Sterol Regulation of Metabolism, Homeostasis, and Development. Annu Rev Biochem. 2011;80: 885–916. doi: 10.1146/annurev-biochem-081308-165917 21495846 PMC3918218

[13] Antebi A. Nuclear receptor signal transduction in C. elegans. WormBook. 2015; 1–49. doi: 10.1895/wormbook.1.64.2 26069085 PMC5402207

[14] Boland S, Schmidt U, Zagoriy V, Sampaio JL, Fritsche RF, Czerwonka R, et al. Phosphorylated glycosphingolipids essential for cholesterol mobilization in Caenorhabditis elegans. Nat Chem Biol. 2017. doi: 10.1038/nchembio.2347 28369040

[15] Galles C, Prez GM, Penkov S, Boland S, Porta EOJ, Altabe SG, et al. Endocannabinoids in Caenorhabditis elegans are essential for the mobilization of cholesterol from internal reserves. Sci Rep. 2018;8: 1–12. doi: 10.1038/s41598-018-24925-8 29686301 PMC5913221

[16] Jie Li, Gemma Brown, Michael Ailion SL and JHT. NCR-1 and NCR-2, the C. elegans homologs of the human Niemann-Pick type C1 disease protein, function upstream of DAF-9 in the dauer formation pathways. Development. 2004;131: 5741–5752. doi: 10.1242/dev.01408 15509773

[17] Pastuhov SI, Matsumoto K, Hisamoto N. Endocannabinoid signaling regulates regenerative axon navigation in Caenorhabditis elegans via the GPCRs NPR-19 and NPR-32. Genes to Cells. 2016;21: 696–705. doi: 10.1111/gtc.12377 27193416

[18] Oakes MD, Law WJ, Clark T, Bamber BA, Komuniecki R. Cannabinoids Activate Monoaminergic Signaling to Modulate Key *C*. *elegans* Behaviors. J Neurosci. 2017;37: 2859–2869. doi: 10.1523/JNEUROSCI.3151-16.2017 28188220 PMC5354331

[19] Chen AL, Lum KM, Lara-Gonzalez P, Ogasawara D, Cognetta AB, To A, et al. Pharmacological convergence reveals a lipid pathway that regulates C. elegans lifespan. Nat Chem Biol. 2019;15: 453–462. doi: 10.1038/s41589-019-0243-4 30911178 PMC6548519

[20] Li X, Shen L, Hua T, Liu ZJ. Structural and Functional Insights into Cannabinoid Receptors. Trends Pharmacol Sci. 2020;41: 665–677. doi: 10.1016/j.tips.2020.06.010 32739033

[21] Hua T, Vemuri K, Nikas SP, Laprairie RB, Wu Y, Qu L, et al. Crystal structures of agonist-bound human cannabinoid receptor CB 1. Nature. 2017;547: 468–471. doi: 10.1038/nature23272 28678776 PMC5793864

[22] Li X, Hua T, Vemuri K, Ho J, Wu Y, Wu L, et al. Crystal Structure of the Human Cannabinoid Receptor CB2. Cell. 2019;176: 459–467. doi: 10.1016/j.cell.2018.12.011 30639103 PMC6713262

[23] Muller C, Morales P, Reggio PH. Cannabinoid ligands targeting TRP channels. Front Mol Neurosci. 2019;11: 1–15. doi: 10.3389/fnmol.2018.00487 30697147 PMC6340993

[24] Tobin DM, Madsen DM, Kahn-Kirby A, Peckol EL, Moulder G, Barstead R, et al. Combinatorial expression of TRPV channel proteins defines their sensory functions and subcellular localization in C. elegans neurons. Neuron. 2002;35: 307–318. doi: 10.1016/s0896-6273(02)00757-2 12160748

[25] Bargmann CI. Chemosensation in C. elegans. WormBook. 2006; 1–29. doi: 10.1895/wormbook.1.123.1 18050433 PMC4781564

[26] Zhang S, Sokolchik I, Blanco G, Sze JY. Caenorhabditis elegans TRPV ion channel regulates 5HT biosynthesis in chemosensory neurons. Development. 2004;131: 1629–1638. doi: 10.1242/dev.01047 14998926

[27] Oakes M, Law WJ, Komuniecki R. Cannabinoids stimulate the TRP channel-dependent release of both serotonin and dopamine to modulate behavior in C. elegans. J Neurosci. 2019;39: 4142–4152. doi: 10.1523/JNEUROSCI.2371-18.2019 30886012 PMC6529862

[28] Kahn-Kirby AH, Bargmann CI. TRP channels in C. elegans. Annu Rev Physiol. 2006;68: 719–736. doi: 10.1146/annurev.physiol.68.040204.100715 16460289

[29] Ohnishi K, Saito S, Miura T, Ohta A, Tominaga M, Sokabe T, et al. OSM-9 and OCR-2 TRPV channels are accessorial warm receptors in Caenorhabditis elegans temperature acclimatisation. Sci Rep. 2020;10: 1–14. doi: 10.1038/s41598-020-75302-3 33122746 PMC7596061

[30] Benham CD, Davis JB, Randall AD. Vanilloid and TRP channels: A family of lipid-gated cation channels. Neuropharmacology. 2002;42: 873–888. doi: 10.1016/s0028-3908(02)00047-3 12069898

[31] Reis Rodrigues P, Kaul TK, Ho J-H, Lucanic M, Burkewitz K, Mair WB, et al. Synthetic Ligands of Cannabinoid Receptors Affect Dauer Formation in the Nematode Caenorhabditis elegans. Genes|Genomes|Genetics. 2016;6: 1695–1705. doi: 10.1534/g3.116.026997 27172180 PMC4889665

[32] Ravil R. Petrova, Lindsay Knight, Shao-Rui Chen, Jim Wager-Miller, Steven W. McDaniel, Fanny Diaz, Francis Barth, Hui-Lin Pan, Ken Mackie, Claudio N. Cavasotto and PD. Mastering tricyclic ring systems for desirable functional cannabinoid activity. Eur J Med Chem. 2013;69: 881–907. doi: 10.1016/j.ejmech.2013.09.038 24125850 PMC3909471

[33] Bladen C, McDaniel SW, Gadotti VM, Petrov RR, Berger ND, Diaz P, et al. Characterization of Novel Cannabinoid Based T-Type Calcium Channel Blockers with Analgesic Effects. ACS Chem Neurosci. 2014;6: 277–287. doi: 10.1021/cn500206a 25314588 PMC4372069

[34] Steger KA, Shtonda BB, Thacker C, Snutch TP, Avery L. The C. elegans T-type calcium channel CCA-1 boosts neuromuscular transmission. J Exp Biol. 2005. doi: 10.1242/jeb.01616 15914662 PMC1382270

[35] Magner DB, Wollam J, Shen Y, Hoppe C, Li D, Latza C, et al. The NHR-8 nuclear receptor regulates cholesterol and bile acid homeostasis in C. elegans. Cell Metab. 2013;18: 212–224. doi: 10.1016/j.cmet.2013.07.007 23931753 PMC3909615

[36] Taghibiglou C, Martin HGS, Rose JK, Ivanova N, Lin CHC, Lau HL, et al. Essential role of SBP-1 activation in oxygen deprivation induced lipid accumulation and increase in body width/length ratio in Caenorhabditis elegans. FEBS Lett. 2009;583: 831–834. doi: 10.1016/j.febslet.2009.01.045 19187779

[37] Lee D, Jeong DE, Son HG, Yamaoka Y, Kim H, Seo K, et al. SREBP and MDT-15 protect C. Elegans from glucose-induced accelerated aging by preventing accumulation of saturated fat. Genes Dev. 2015;29: 2490–2503. doi: 10.1101/gad.266304.115 26637528 PMC4691952

[38] Gévry N, Schoonjans K, Guay F, Murphy BD. Cholesterol supply and SREBPs modulate transcription of the niemann-pick C-1 gene in steroidogenic tissues. J Lipid Res. 2008;49: 1024–1033. doi: 10.1194/jlr.M700554-JLR200 18272928

[39] Hannich JT, Entchev E V., Mende F, Boytchev H, Martin R, Zagoriy V, et al. Methylation of the Sterol Nucleus by STRM-1 Regulates Dauer Larva Formation in Caenorhabditis elegans. Dev Cell. 2009;16: 833–843. doi: 10.1016/j.devcel.2009.04.012 19531354

[40] Saifee O, Wei L, Nonet ML. The Caenorhabditis elegans unc-64 locus encodes a syntaxin that interacts genetically with synaptobrevin. Mol Biol Cell. 1998;9: 1235–1252. doi: 10.1091/mbc.9.6.1235 9614171 PMC25346

[41] Ann K, Kowalchyk JA, Loyet KM, Martin TFJ. Novel Ca2+-binding protein (CAPS) related to UNC-31 required for Ca2+-activated exocytosis. J Biol Chem. 1997;272: 19637–19640. doi: 10.1074/jbc.272.32.19637 9289490

[42] Ailion M, Inoue T, Weaver CI, Holdcraft RW, Thomas JH. Neurosecretory control of aging in Caenorhabditis elegans. Proc Natl Acad Sci U S A. 1999;96: 7394–7397. doi: 10.1073/pnas.96.13.7394 10377425 PMC22096

[43] Berwin B, Floor E, Martin TFJ. CAPS (Mammalian UNC-31) Protein Localizes to Membranes Involved in Dense-Core Vesicle Exocytosis. Neuron. 1998;21: 137–145. doi: 10.1016/s0896-6273(00)80521-8 9697858

[44] Leinwand SG, Chalasani SH. From genes to circuits and behaviors: Neuropeptides expand the coding potential of the nervous system. Worm. 2014;3: e27730. doi: 10.4161/worm.27730 25254145 PMC4165544

[45] Lee BH, Ashrafi K. A TRPV channel modulates C. elegans neurosecretion, larval starvation survival, and adult lifespan. PLoS Genet. 2008;4: 1–14. doi: 10.1371/journal.pgen.1000213 18846209 PMC2556084

[46] Yu Y, Wang L, Jiu Y, Zhan Y, Liu L, Xia Z, et al. HID-1 is a novel player in the regulation of neuropeptide sorting. Biochem J. 2011;434: 383–390. doi: 10.1042/BJ20110027 21250940

[47] Di Marzo V, Bifulco M, De Petrocellis L. The endocannabinoid system and its therapeutic exploitation. Nat Rev Drug Discov. 2004;3: 771–784. doi: 10.1038/nrd1495 15340387

[48] Cristino L, Bisogno T, Di Marzo V. Cannabinoids and the expanded endocannabinoid system in neurological disorders. Nat Rev Neurol. 2020;16: 9–29. doi: 10.1038/s41582-019-0284-z 31831863

[49] Clarke TL, Johnson RL, Simone JJ, Carlone RL. The endocannabinoid system and invertebrate neurodevelopment and regeneration. Int J Mol Sci. 2021;22: 1–24. doi: 10.3390/ijms22042103 33672634 PMC7924210

[50] Lucanic M, Held JM, Vantipalli MC, Klang IM, Graham JB, Gibson BW, et al. N-acylethanolamine signalling mediates the effect of diet on lifespan in Caenorhabditis elegans. Nature. 2011;473: 226–229. doi: 10.1038/nature10007 21562563 PMC3093655

[51] Hammarlund M, Watanabe S, Schuske K, Jorgensen EM. CAPS and syntaxin dock dense core vesicles to the plasma membrane in neurons. J Cell Biol. 2008;180: 483–491. doi: 10.1083/jcb.200708018 18250196 PMC2234227

[52] Kobayashi T, Beuchat MH, Lindsay M, Frias S, Palmiter RD, Sakuraba H, et al. Late endosomal membranes rich in lysobisphosphatidic acid regulate cholesterol transport. Nat Cell Biol. 1999;1: 113–118. doi: 10.1038/10084 10559883

[53] Dainese E, Oddi S, Maccarrone M. Interaction of Endocannabinoid Receptors with Biological Membranes. Curr Med Chem. 2010;17: 1487–1499. doi: 10.2174/092986710790980087 20166920

[54] Di Scala C, Fantini J, Yahi N, Barrantes FJ, Chahinian H. Anandamide revisited: How cholesterol and ceramides control receptor-dependent and receptor-independent signal transmission pathways of a lipid neurotransmitter. Biomolecules. 2018;8. doi: 10.3390/biom8020031 29789479 PMC6022874

[55] de Boer JF, Kuipers F, Groen AK. Cholesterol Transport Revisited: A New Turbo Mechanism to Drive Cholesterol Excretion. Trends Endocrinol Metab. 2018;29: 123–133. doi: 10.1016/j.tem.2017.11.006 29276134

[56] Van Voorhies WA, Ward S. Genetic and environmental conditions that increase longevity in Caenorhabditis elegans decrease metabolic rate. Proc Natl Acad Sci U S A. 1999;96: 11399–11403. doi: 10.1073/pnas.96.20.11399 10500188 PMC18045

[57] Osei-Hyiaman D, DePetrillo M, Pacher P, Liu J, Radaeva S, Bátkai S, et al. Endocannabinoid activation at hepatic CB 1 receptors stimulates fatty acid synthesis and contributes to diet-induced obesity. J Clin Invest. 2005;115: 1298–1305. doi: 10.1172/JCI23057 15864349 PMC1087161

[58] Murphy CT, Hu PJ. Insulin/insulin-like growth factor signaling in C. elegans. WormBook. 2013; 1–43. doi: 10.1895/wormbook.1.164.1 24395814 PMC4780952

[59] Kahn-Kirby AH, Dantzker JLM, Apicella AJ, Schafer WR, Browse J, Bargmann CI, et al. Specific polyunsaturated fatty acids drive TRPV-dependent sensory signaling in vivo. Cell. 2004;119: 889–900. doi: 10.1016/j.cell.2004.11.005 15607983

[60] Brenner S. The genetics of Caenorhabditis elegans. Genetics. 1974;77: 71–94. doi: 10.1093/genetics/77.1.71 4366476 PMC1213120

[61] Folch J, Lees M, Slone stanley GH. A simple method for the isolation and purification of total lipides from animal tissues. J Biol Chem. 1957;226: 497–509. doi: 10.1016/s0021-9258(18)64849-5 13428781

[62] Chalasani SH, Chronis N, Tsunozaki M, Gray JM, Ramot D, Goodman MB, et al. Dissecting a circuit for olfactory behaviour in Caenorhabditis elegans. Nature. 2007;450: 63–70. doi: 10.1038/nature06292 17972877

[63] Chronis N, Zimmer M, Bargmann CI. Microfluidics for in vivo imaging of neuronal and behavioral activity in Caenorhabditis elegans. Nat Methods. 2007;4: 727–731. doi: 10.1038/nmeth1075 17704783

[64] Arthur D. Edelstein, Mark A. Tsuchida, Nenad Amodaj, Henry Pinkard, Ronald D. Vale and NS. Advanced methods of microscope control using μManager software. J Biol Methods. 2015;1: 1–18. doi: 10.14440/jbm.2014.36 25606571 PMC4297649

