# Supplementary figures and images for: Cannabinoids activate the insulin pathway to modulate mobilization of cholesterol in C. elegans
Source: PLoS Genet. 2022 Nov 8;18(11):e1010346. doi: 10.1371/journal.pgen.1010346 (PMC9674138; doi:10.1371/journal.pgen.1010346)

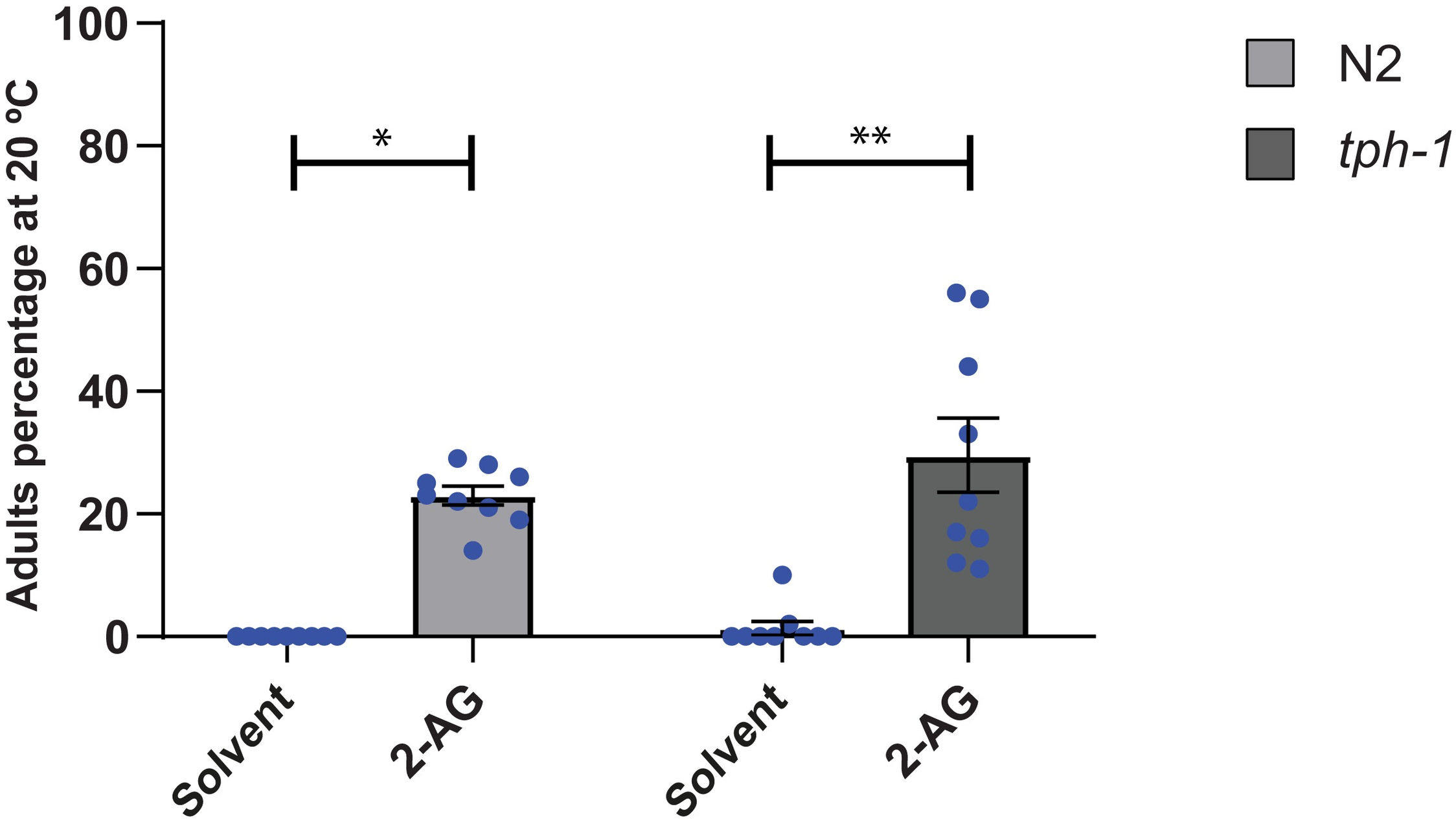

Supplement: S1 Fig — (A) N2 and tph-1 were grown for two generations in media with 0 μg/ml cholesterol at 20°C. Mann-Whitney rank sum test, *p < 0.001. t-test, **p < 0.001. All values are from n = 3 independent experiments shown as Mean ± SEM. N2 is the C. elegans wild-type strain. (TIF) [file pgen.1010346.s001.tif]

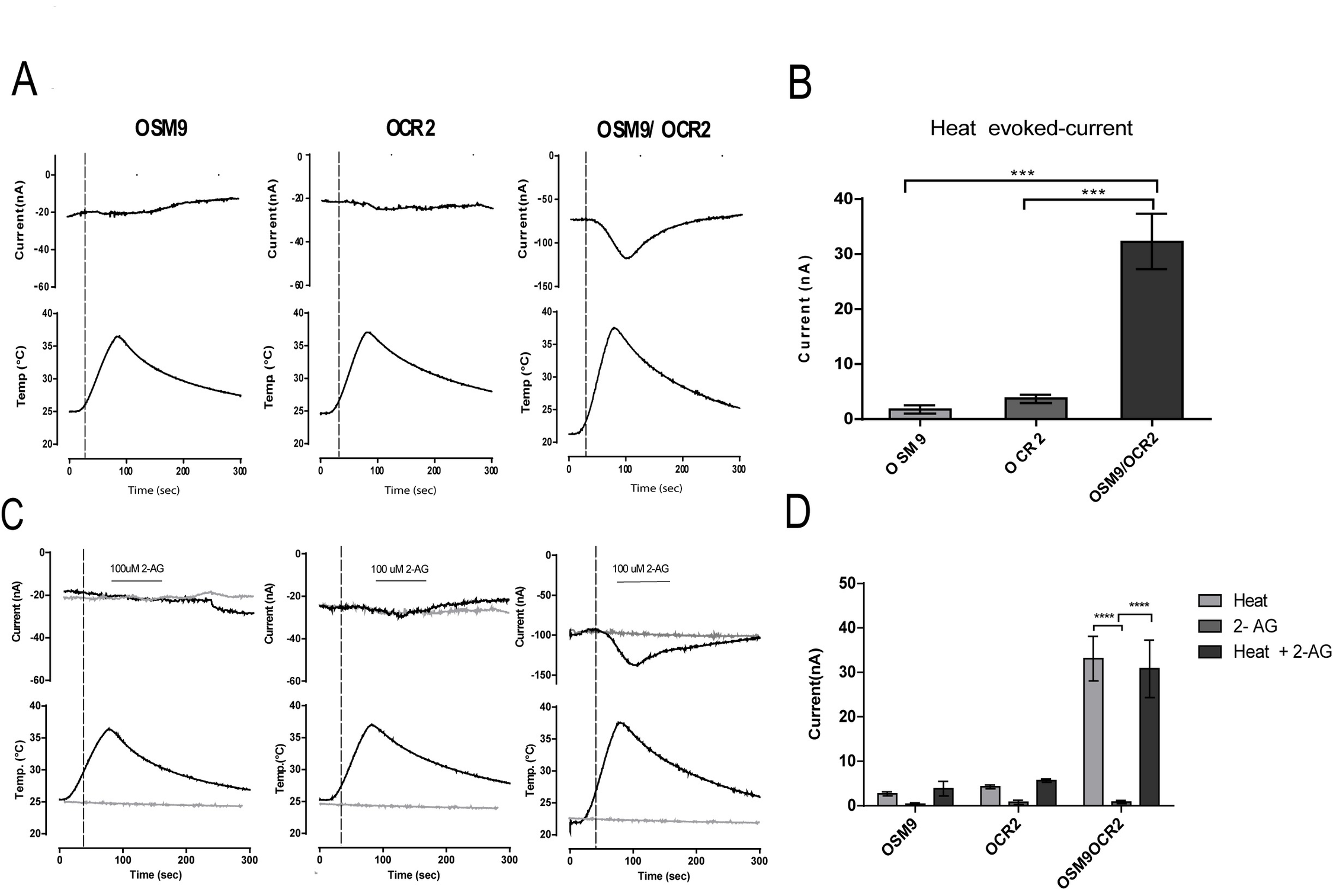

Supplement: S2 Fig — (A) Representative traces of responses (above) and temperature (below) in Xenopus oocytes injected with cRNAs encoding OSM-9, OCR-2, OSM-9/OCR-2. (B) Mean ± SEM of heat evoked currents. Amplitudes were calculated by measuring the differences between the peak inward currents and baseline marked with dotted lines (***p = 0.0001, n ≥ 5 per group, ANOVA followed by a Bonferroni’s multi-comparison test). (C) Representative traces of responses to 100 μM 2-AG either at room temperature (grey traces) or during a temperature ramp in oocytes expressing OSM-9, OCR-2 or OSM-9/OCR-2. (D) Mean ± SEM of current amplitudes of responses to temperature, 100 μM 2-AG and temperature plus 100 μM 2-AG in oocytes injected with either OSM-9, OCR-2 or OSM-9/OCR-2 (**** p < 0.0001, n ≥ 3 oocytes per group, two-way ANOVA followed by a Bonferroni multi- comparison test). (TIF) [file pgen.1010346.s002.tif]

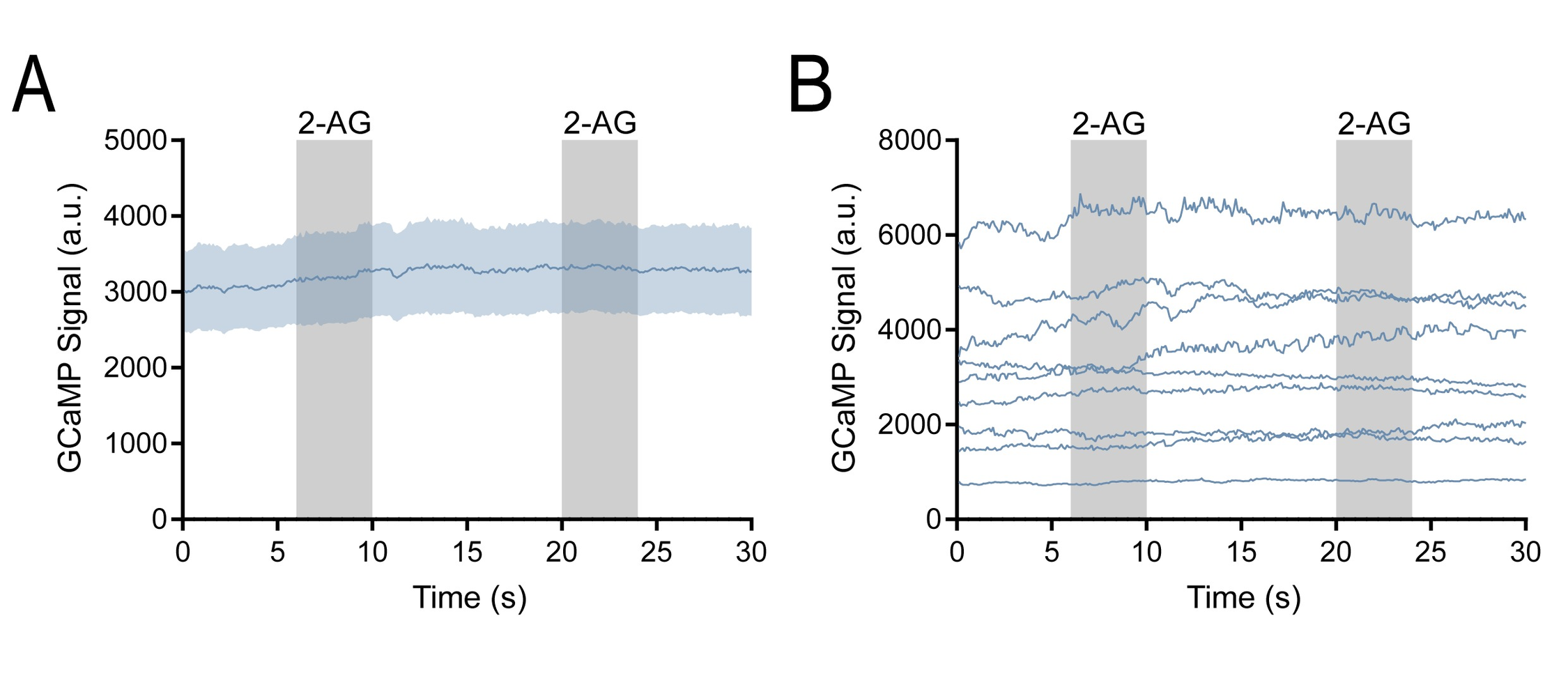

Supplement: S3 Fig — Calcium imaging traces of animals expressing GCaMP6 in the ASH (xuEx1978 [Psra-6::Gcamp6(f), Psra-6::DsRed]) during exposure to 2-AG. Average responses for all animals (A) are indicated by the dark blue line and the shaded area represents the SEM. The same results are shown as individual traces for each animal (B). The dark grey bars indicate 4 second exposure to buffer containing 100 mM 2-AG. (TIF) [file pgen.1010346.s003.tif]

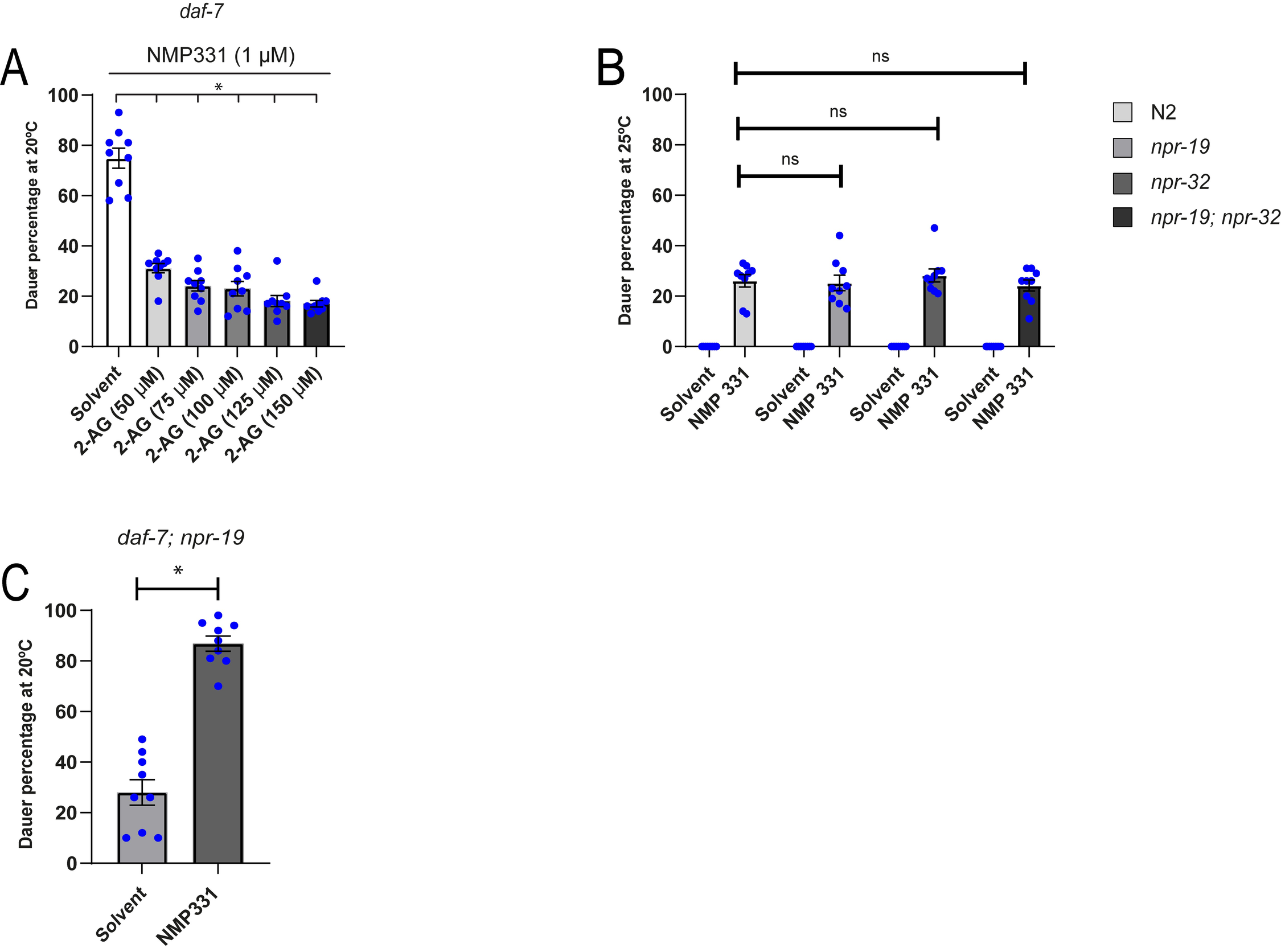

Supplement: S4 Fig — (A) 2-AG antagonizes the effect of 1 μM of NMP331 in a daf-7 worm at 20°C. All pairwise are multiple comparison procedures (Holm-Sidak method), *p < 0.001. All values are from n = 3 independent experiments shown as Mean ± SEM. (B) N2 and npr animals undergo a dauer-like arrest induced by NMP331 in the first generation when grown in cholesterol free medium. The concentration of NMP331 was 50 μM. ns = not significant (C) NMP331 enhances dauer formation in daf-7; npr-19 animals in media with cholesterol 13 μM. Mann-Whitney rank sum test, *p < 0.001. All values are from n = 3 independent experiments are shown as Mean ± SEM. (TIF) [file pgen.1010346.s004.tif]

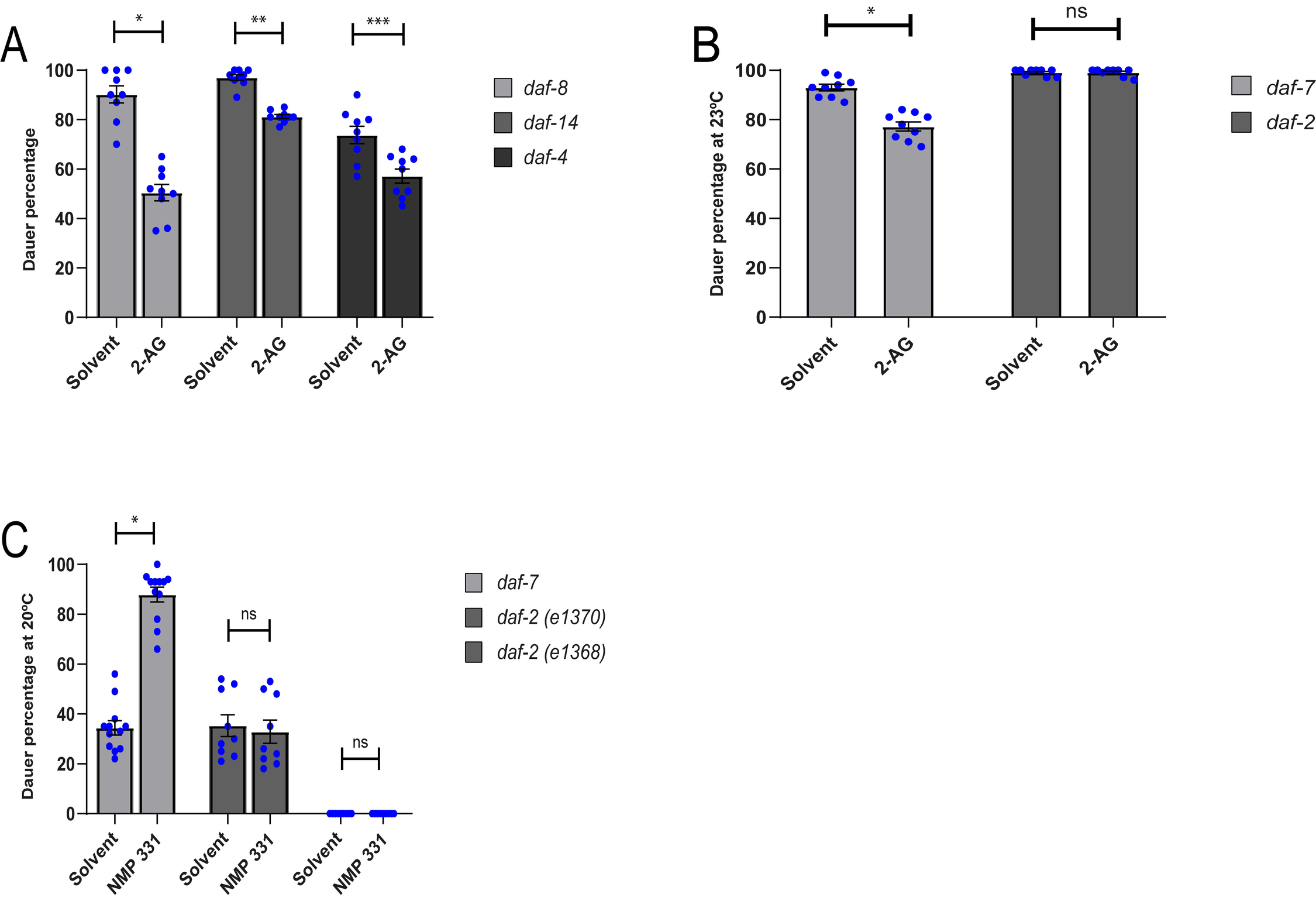

Supplement: S5 Fig — (A). Daf-4 was grown at 20°C while daf-14 and daf-8 were grown at 25°C under normal dietary cholesterol (13 μM). t-test, *p < 0.001. **p < 0.001. ***p < 0.002. All values are from n = 3 independent experiments shown as Mean ± SEM. (B) daf-7 and daf-2 were grown at 23°C in a sterol-free media during one generation. t-test, *p < 0.001. All values are from n ≥ 3 independent experiments shown as Mean ± SEM. ns = not significant. (C) NMP331 does not enhance the daf-c phenotype of daf-2 mutants. Mann-Whitney rank sum test, *p < 0.001. All values are from n ≥ 3 independent experiments shown as Mean ± SEM. ns = not significant. (TIF) [file pgen.1010346.s005.tif]

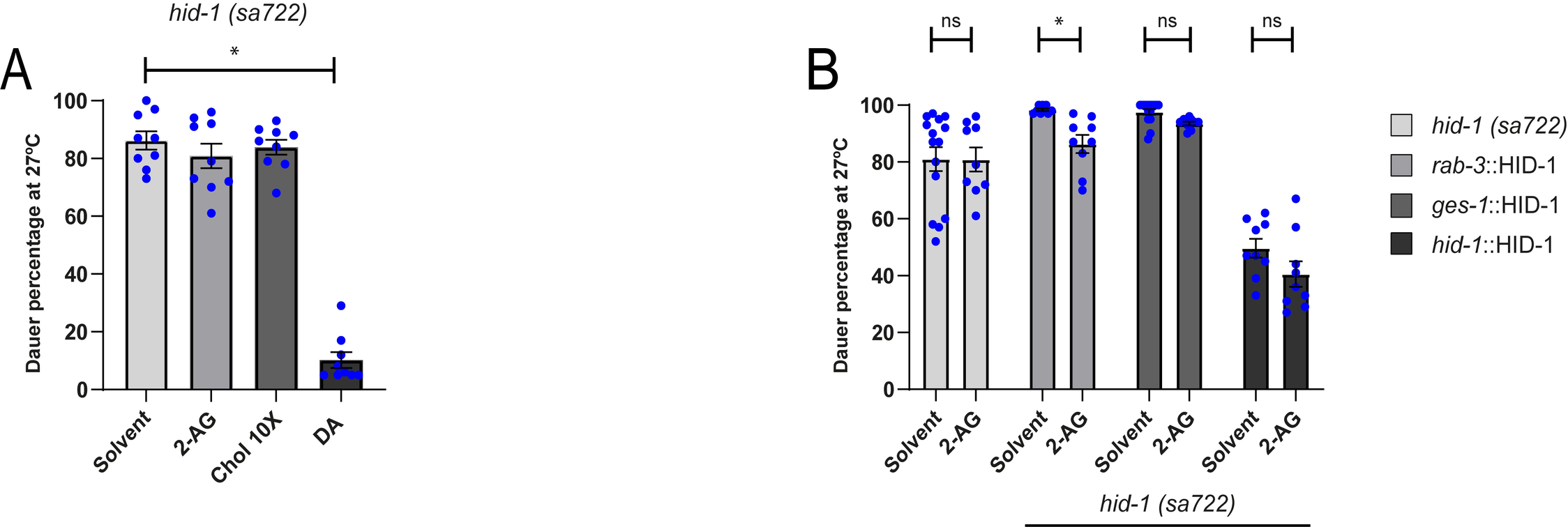

Supplement: S6 Fig — (A) Hid-1 is required for 2-AG dependent mobilization of cholesterol. Worms were grown at 27°C under normal dietary cholesterol. All pairwise are multiple comparison procedures (Holm-Sidak method), *p < 0.05. All values are from n = 3 independent experiments shown as Mean ± SEM. ns = not significant. (B) HID-1 expression in neurons in a hid-1 background restores the 2-AG-dependent mobilization of cholesterol. Worms were grown at 27°C under normal dietary cholesterol. Mann-Whitney rank sum test, *p < 0.001. All values are from n ≥ 3 independent experiments shown as Mean ± SEM. ns = not significant. (TIF) [file pgen.1010346.s006.tif]

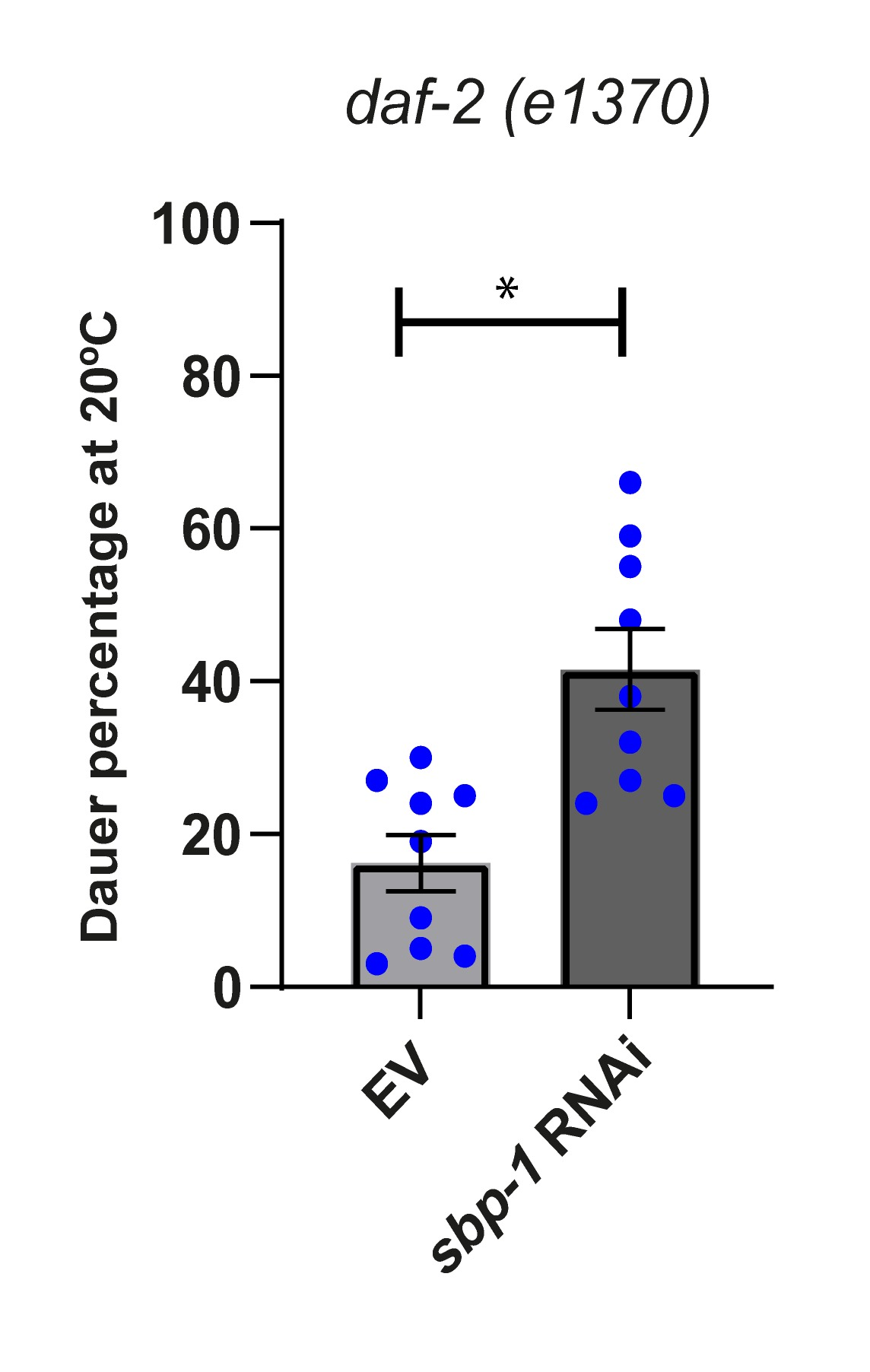

Supplement: S7 Fig — daf-2 was grown in 13 μM cholesterol at 20°C. Mann-Whitney rank sum test, *p < 0.005. All values are from n = 3 independent experiments shown as Mean ± SEM. (TIF) [file pgen.1010346.s007.tif]
