# Supplementary material for: Cannabinoids activate the insulin pathway to modulate mobilization of cholesterol in C. elegans
Source: PLoS Genet. 2022 Nov 8;18(11):e1010346. doi: 10.1371/journal.pgen.1010346 (PMC9674138; doi:10.1371/journal.pgen.1010346)
Supplement: S1 Table — (DOCX) [file pgen.1010346.s008.docx]

**S1 Table**.

| **Genotype** | **Gene Function** | **Treatment** | **Adults Percentage*** | **SEM** | **Number of Experiments** |
| --- | --- | --- | --- | --- | --- |
|  |  |  |  |  |  |
| N2 | wild-type | Solvent | 0 | 0 | 4 |
|  |  | 2-AG (50 µM) | 23.5 | 2.7 | 4 |
| *ser-1* | Is an ortholog of human 5-hydroxytryptamine receptor 2B | Solvent | 1 | 0.66 | 3 |
|  |  | 2-AG (50 µM) | 46 | 7.53 | 3 |
| *ser-2* | Exhibits tyramine receptor activity | Solvent | 0 | 0 | 3 |
|  |  | 2-AG (50 µM) | 53 | 5.91 | 3 |
| *ser-4* | Is an ortholog of human 5-hydroxytryptamine receptor 1A | Solvent | 0 | 0 | 3 |
|  |  | 2-AG (50 µM) | 31.88 | 3.97 | 3 |
| *ser-7* | Is an ortholog of human 5-hydroxytryptamine receptor 7 | Solvent | 0 | 0 | 3 |
|  |  | 2-AG (50 µM) | 47.33 | 5.92 | 3 |
| *gar-1* | Exhibits G protein-coupled acetylcholine receptor activity. | Solvent | 0 | 0 | 3 |
|  |  | 2-AG (50 µM) | 32.55 | 3.48 | 3 |
| *gar-2* | Exhibits G protein-coupled acetylcholine receptor activity | Solvent | 0,5 | 0.5 | 2 |
|  |  | 2-AG (50 µM) | 17.33 | 1.81 | 2 |
| *gar-3* | Is an ortholog of human cholinergic receptor muscarinic 1; 3; and 5 | Solvent | 0 | 0 | 2 |
|  |  | 2-AG (50 µM) | 37 | 8.73 | 2 |
| *dop-1* | Is an ortholog of human dopamine receptor D1 and D5 | Solvent | 0 | 0 | 2 |
|  |  | 2-AG (50 µM) | 31.5 | 2.39 | 2 |
| *dop-2* | Is an ortholog of human dopamine receptor D2 and D3 | Solvent | 4 | 1.73 | 2 |
|  |  | 2-AG (50 µM) | 46.5 | 9.17 | 2 |
| *dop-4* | Exhibits dopamine neurotransmitter receptor activity. | Solvent | 0 | 0 | 3 |
|  |  | 2-AG (50 µM) | 16.77 | 4.59 | 3 |
| *mod-1* | Exhibits G protein-coupled serotonin receptor activity and chloride channel activity. | Solvent | 0 | 0 | 2 |
|  |  | 2-AG (50 µM) | 68.66 | 5.07 | 2 |
| *tyra-3* | Is predicted to have G protein-coupled serotonin receptor activity and octopamine receptor activity | Solvent | 0 | 0 | 2 |
|  |  | 2-AG (50 µM) | 29.16 | 2.8 | 2 |
| *gnrr-1* | Is an ortholog of human GNRHR (gonadotropin releasing hormone receptor) | Solvent | 0 | 0 | 2 |
|  |  | 2-AG (50 µM) | 32.5 | 6.18 | 2 |
| *npr-11* | Is predicted to have neuropeptide Y receptor activity | Solvent | 0 | 0 | 2 |
|  |  | 2-AG (50 µM) | 68 | 5.73 | 2 |
| *npr-16* | Is predicted to have G protein-coupled receptor activity and peptide binding activity. | Solvent | 2.75 | 2.12 | 2 |
|  |  | 2-AG (50 µM) | 23.16 | 5.3 | 2 |
| *npr-19* | Is predicted to have G protein-coupled receptor activity. | Solvent | 0 | 0 | 3 |
|  |  | 2-AG (50 µM) | 15.22 | 2.05 | 3 |
| *npr-24* | Is an ortholog of human somatostatin receptor 5 | Solvent | 0 | 0 | 2 |
|  |  | 2-AG (50 µM) | 51.16 | 6.53 | 2 |
| *npr-32* | Is predicted to have G protein-coupled receptor activity. | Solvent | 0 | 0 | 3 |
|  |  | 2-AG (50 µM) | 25.11 | 4.88 | 3 |
| *npr-35* | Is predicted to have G protein-coupled receptor activity | Solvent | 0 | 0 | 2 |
|  |  | 2-AG (50 µM) | 55.33 | 7.99 | 2 |
| *lbp-1* | Is predicted to have lipid binding activity. | Solvent | 0 | 0 | 2 |
|  |  | 2-AG (50 µM) | 21 | 2.92 | 2 |
| *octr-1* | Is an ortholog of human adrenoceptor alpha 2A. Exhibits octopamine receptor activity | Solvent | 0 | 0 | 3 |
|  |  | 2-AG (50 µM) | 42.77 | 7.13 | 3 |
| *ckr-2* | Is an ortholog of human cholecystokinin B receptor. Exhibits neuropeptide binding activity | Solvent | 4.33 | 1.58 | 3 |
|  |  | 2-AG (50 µM) | 75.77 | 4.46 | 3 |
| *chup-1* | Is an ortholog of human SID1 transmembrane family member 1 and 2. Exhibits cholesterol binding activity. | Solvent | 0 | 0 | 2 |
|  |  | 2-AG (50 µM) | 20.5 | 3.14 | 2 |

In the fourth column the * corresponds to the average of total experiments performed.
