# Supplementary material for: Cannabinoids activate the insulin pathway to modulate mobilization of cholesterol in C. elegans
Source: PLoS Genet. 2022 Nov 8;18(11):e1010346. doi: 10.1371/journal.pgen.1010346 (PMC9674138; doi:10.1371/journal.pgen.1010346)
Supplement: S2 Table — For details see text. (DOCX) [file pgen.1010346.s009.docx]

**S2 Table.**

| **Gene Name** | **Predictive gene function** | ***daf-7* dauers percentage at 20ºC** †† | **SEM** | **Number of Experiments** | **Stadistical diference vs EV** |
| --- | --- | --- | --- | --- | --- |
|  |  |  |  |  |  |
| Empty Vector | Control | 52.91 | 4.28 | 4 |  |
|  |  |  |  |  |  |
| *daf-16* (positive control*)* | Is an ortholog of human FOXO1; FOXO3 ; and FOXO4. | 35.33 | 4.73 | 3 | **YES*** |
|  |  |  |  |  |  |
| *lrp-1* | LDL receptor related protein 2 | 60.88 | 4.45 | 3 | NO |
|  |  |  |  |  |  |
| *lrp-2* | LDL receptor related protein 1 | 43.88 | 5.73 | 3 | NO |
|  |  |  |  |  |  |
| *ced-1* | Scavenger Receptor ortholog of human MEGF10 | 36.77 | 6.96 | 3 | NO |
|  |  |  |  |  |  |
| *F13H10.3* | Ortholog of human SLC38A9 | 54.16 | 5.47 | 2 | NO |
|  |  |  |  |  |  |
| *mboa-1* | Ortholog of human sterol O-acyltransferase 1 | 62 | 4.98 | 3 | NO |
|  |  |  |  |  |  |
| *abt-2* | Ortholog of human ATP binding cassette subfamily A member 1 | 73.83 | 3.04 | 4 | **YES**** |
|  |  |  |  |  |  |
| *obr-2* | Ortholog of human OSBPL2 (oxysterol binding protein like 2) | 49.5 | 5.44 | 2 | NO |
|  |  |  |  |  |  |
| *obr-3* | Is an ortholog of human OSBPL5 (oxysterol binding protein like 5) | 58.66 | 5.94 | 2 | NO |
|  |  |  |  |  |  |
| *obr-4* | Ortholog of human OSBPL9 (oxysterol binding protein like 9) | 46.66 | 6.29 | 3 | NO |
|  |  |  |  |  |  |
| *rme-2* | LDL receptor related protein 5 like | 57.66 | 5.4 | 2 | NO |
|  |  |  |  |  |  |
| *nceh-1* | Ortholog of human neutral cholesterol ester hydrolase 1 | 78.5 | 3.31 | 4 | **YES***** |
|  |  |  |  |  |  |
| *lbp-1* | Lipid Binding Protein | 66.16 | 3.68 | 4 | NO |
|  |  |  |  |  |  |
| *scp-1* | Is an ortholog of human SCAP | 71.44 | 2.83 | 3 | **YES****** |
|  |  |  |  |  |  |
| *sbp-1* | Is an ortholog of human SREBP-1C | 81.11 | 2.47 | 3 | **YES******* |
|  |  |  |  |  |  |
| *nhr-8* | Is an ortholog of human nuclear receptor subfamily 1 group I member 2 and vitamin D receptor. | 72.44 | 4.17 | 3 | **YES******** |
|  |  |  |  |  |  |

*- t-test, p < 0.05

**- t-test, p < 0.001

***- t-test, p < 0.001

****- t-test, p < 0.005

*****- t-test, p < 0.001

******- t-test, p < 0.01

†- *t-test were performed in SigmaPlot Software*

††- The results provided in the third column correspond to the average of total experiments performed.
